# **Crystal structure of [1,2,4]** **triazolo[4,3-** ***b*** **]pyridazine derivatives as BRD4 bromodomain inhibitors and structure–activity relationship study**

**DOI:** 10.1038/s41598-023-37527-w

**Published:** 2023-07-04

**Authors:** Jung-Hoon Kim, Navin Pandit, Miyoun Yoo, Tae Hyun Park, Ji U Choi, Chi Hoon Park, Kwan-Young Jung, Byung Il Lee

**Affiliations:** 1 Research Institute, National Cancer Center, Goyang, Gyeonggi 10408 Republic of Korea; 2 Department of Cancer Biomedical Science, National Cancer Center Graduate School of Cancer Science and Policy, Goyang, Gyeonggi 10408 Republic of Korea; 3 Department of Medicinal Chemistry and Pharmacology, University of Science and Technology, Daejeon, 34113 Republic of Korea; 4 Therapeutics and Biotechnology Division, Korea Research Institute of Chemical Technology, Daejeon, 34114 Republic of Korea; 5 Department of Anesthesiology, Weill Cornell Medical College, New York, NY 10065 USA

**Keywords:** X-ray crystallography, Structure-based drug design

## Abstract

BRD4 contains two tandem bromodomains (BD1 and BD2) that recognize acetylated lysine for epigenetic reading, and these bromodomains are promising therapeutic targets for treating various diseases, including cancers. BRD4 is a well-studied target, and many chemical scaffolds for inhibitors have been developed. Research on the development of BRD4 inhibitors against various diseases is actively being conducted. Herein, we propose a series of [1,2,4]triazolo[4,3-*b*]pyridazine derivatives as bromodomain inhibitors with micromolar IC_50_ values. We characterized the binding modes by determining the crystal structures of BD1 in complex with four selected inhibitors. Compounds containing [1,2,4] triazolo[4,3-*b*]pyridazine derivatives offer promising starting molecules for designing potent BRD4 BD inhibitors.

## Introduction

The bromodomain and extraterminal (BET) protein families (BRD2, BRD3, BRD4, and BRDT) are proteins with two canonical tandem bromodomains (BD1 and BD2) that recognize acetylated lysine (Kac) at their N termini^1,2^. Until now, the molecular functions of these tandem domains were suggested to be transcriptional^1,3^ and nontranscription-related functions^4–7^. These two bromodomains share high sequence and structure similarity^8,9^. However, slight differences in surface charges and structure in the Kac binding site result in a distinct selectivity in the binding of acetylated histone/nonhistone peptides and induce various biological functions^1,2^.

Among BET family members, bromodomain-containing protein 4 (BRD4) has been extensively studied. As a key components of a transcriptional complex in epigenetic events, BRD4 functions as a reader, and two bromodomains of BRD4 specifically interact with Kac containing the N-terminus of histones^1^. The hydrophobic cavity, which is also called the Kac binding pocket^4,10^, possesses a functional surface that specifically interacts with Kac and has roles in epigenetic readers by forming a transcriptional multiprotein complex^1,8,11^. BRD4 is widely expressed in the body and is a promising therapeutic target for treating cancers^10,12–15^ and noncancer diseases^16,17^, considering its disease-related changes in expression level^18–20^.

In 2010, two BRD4 inhibitors were introduced, JQ1( +) (hereafter, JQ1) and GSK525762A (I-BET762), which have similar core chemical scaffolds^3,21^. After these discoveries, extensive efforts to discover BRD4 BD1/BD2 inhibitors with high efficacy and specificity have been conducted. Recently, the development of small molecule inhibitors of BRD4 has involved the following main strategies: blockade molecules (selective BET inhibitor; BETi^2,22,23^ and pan BET inhibitor; pan BETi^24^) and degraders (degrader BET; dBET^25,26^). Many reports have shown that pan BETis, such as JQ1 and iBET762, exhibited no selectivity between BD1 and BD2, but BD1-selective (GSK778) or BD2-selective (GSK046 and ABBV-744) BETis showed significant IC_50_ value differences between BD1 and BD2^2,9,22,23^. In addition, recent studies have shown that selective targeting of BD1 and BD2 of BET proteins results in different treatment effectiveness. For example, whereas a BD1-selective inhibitor (GSK778) showed similar phenocopies of pan BETis in cancers, a BD2-selective inhibitor (GSK046) showed better effectiveness in inflammatory and autoimmune diseases^2^. Another report showed that BD2-selective BET family inhibitors exhibited good efficacies in treating prostate cancer^22^. Furthermore, targeted protein degraders or proteolysis-targeting chimeras (PROTACs; dBET1 and ARV-771) utilizing small molecules of BRD4 BETis (JQ1, I-BET762) have shown promising treatment efficacies in many types of cancers and immune-related diseases^25,26^. Chemical structures of representative examples of each type of inhibitors are shown in Supplementary Fig. S1.

Extensive structural studies of BRD4 bromodomains in complex with Kac peptides or various inhibitors have revealed critical residues of BD1/BD2 that participate in protein or ligand binding^3,8,27–29^. They also proposed the binding modes of ligands and contributed to structure-based inhibitor discoveries.

In this study, we present a chemical scaffold of [1,2,4]triazolo[4,3-*b*]pyridazine derivatives as bromodomain inhibitors. As an extension of previous works^30,31^, we discovered a hit molecule by the AlphaScreen assay for determining biological activity. More than thirty derivatives of the hit compound were assessed by in vitro assay, and several inhibitors with micromolar IC_50_ values were found. In addition, we comprehensibly analyzed our BD1–inhibitor complex structures and other ligand-bound BD1 structures deposited in the Protein Data Bank (PDB). Our results provide structural insight into the development of efficient inhibitors of BRD4 BD1/BD2 through structure-guided strategies.

## Results and discussion

### Analysis of the ligand binding cavity with BRD4 BD1 structures

**Figure 1:**
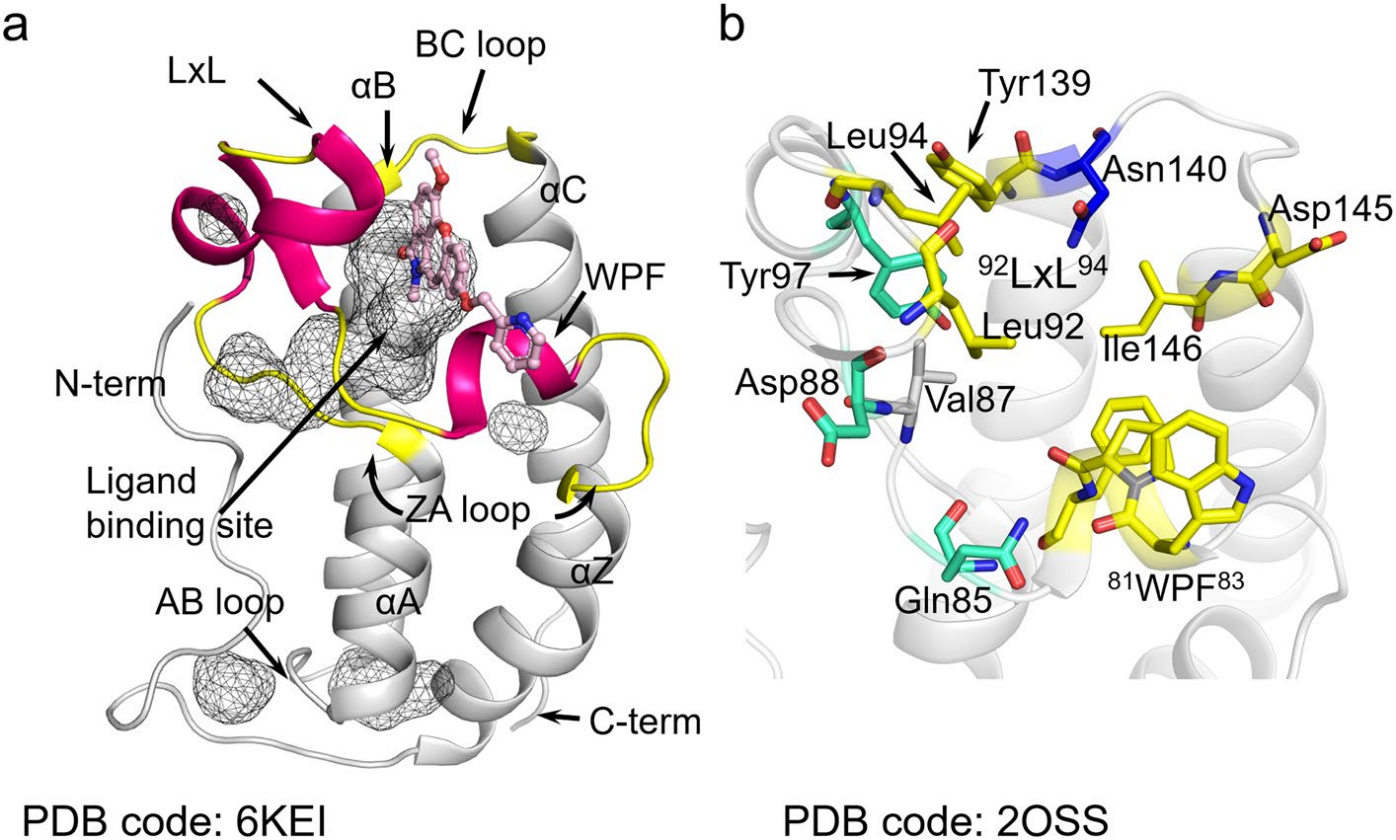
The overall structure of BRD4 BD1. 6KEI is aristoyagonine derivative-bound BRD4 BD1, and 2OSS is apo-BRD4 BD1. (**a**) Each secondary structure element is labeled in the figure. The ligand binding site of BD1 was illustrated by the cavity mode suite using the program PyMOL (Schrödinger, New York, NY, USA). In the ZA loop (yellow), three helices are illustrated in magenta. The compound (pink) was bound in the cavity. (**b**) Key residues participating in Kac peptide or inhibitor binding are depicted in the apo form of BRD4 BD1. Most of the key residues are positioned at the ZA loop and BC loop (blue: common hydrogen bond-forming residue, yellow: common hydrophobic interacting residues, cyan: additional ligandable residues in previous reports^30,31^). The figures were drawn using the program PyMOL version 2.0 (Schrödinger, New York, NY, USA, https://www.schrodinger.com/products/pymol).

We comprehensively compared deposited structures in the PDB and determined the key ligand interacting residues by using the Protein‒Ligand Interaction Profiler (PLIP) server, which detect and list the various types of interactions between protein and ligand^32,33^. BD1 has four α helices (αZ, αA, αB, and αC) as the skeletal backbone, and they are linked with three loops named the ZA, AB, and BC loops (Fig. 1a). The Kac and inhibitor binding site (ligand binding site) of BD1 is formed by ZA and BC loops, and most interacting residues reside on those loops (yellow and magenta in Fig. 1a,b; the key residues are illustrated as sticks). A characteristic WPF motif (^81^WPF^83^), which is important for ligand or peptide binding, resides in the ZA loop (Fig. 1).

Crystal structures of the substrate histone peptide-bound BD1 showed structural insight for binding drugs (Supplementary Fig. S2). In the monoacetylated lysine (mo-Kac)-bound BD1 structure (PDB code: 3JVK), the long sidechain of the acetylated lysine of the Kac peptide mostly inserted into the deep cavity and interacted with the protein through hydrophobic interactions (Supplementary Fig. S2a). In particular, the terminal acetyl group is critical for interaction. As readers of lysine acetylation, the di-acetylated lysine (di-Kac) histone tail exhibits a higher affinity than that of mo-Kac, and the di-Kac peptide-bound cocrystal structures (PDB code: 3UVW, 3UVX, and 3UVY) are also informative in characterizing the ligand binding mode in BRD4 BD1^1,8^. The first Kac (Kac1) in the di-Kac peptide showed a similar binding mode to the mo-Kac peptide, but additional interactions can strengthen the interaction (Supplementary Fig. S2b–g). In detail, the Kac1 formed a hydrogen bond between the carbonyl of the acetyl group of Kac and the amine of Asn140 in the BC loop, and multiple hydrophobic interactions with some hydrophobic residues (Phe83, Val87, Leu92, and/or Leu94) were also found (Supplementary Fig. S2b,d,f). The second Kac (Kac2) usually forms extensive hydrophobic interactions with residues in the ZA loop, particularly, WPF motif. In some structures, Kac2 also make interactions with Asp145 and Ile146 (Supplementary Fig. S2d,f). Additionally, the other parts of the di-Kac peptides, including mainchain, forms multiple hydrogen bonds with residues in the BC loop (Supplementary Fig. S2c,e,g). It is interesting that the two individual Kac (Kac1, Kac2) residues are usually connected by several conserved water molecules near the WPF motif (Supplementary Fig. S3).

In the ZA loop, there are three 3_10_ helices that can easily be unwound (Fig. 1). Two of them contain many ligand-interacting residues. The first turn is the conserved WPF motif, which contributes hydrophobic interactions with other ligands. The second helix possesses another conserved sequence, which contains two conserved leucine residues (^92^LxL^94^; for the x, Asn in BD1, Gly in BD2). These two leucine residues also participate in hydrophobic interactions with ligands. Taken together, the results indicate that hydrophobic residues in the WPF and LxL motifs form hydrophobic interactions with the substrates and inhibitors. In addition to these common key residues, the Val87, Tyr139, Asp145, and Ile146 residues also participate in forming hydrophobic interactions with ligands in BD1. Asn140 is an important residue that forms a hydrogen bond with the ligand. Additionally, in our recent reports, benzo-oxepinoindol derivative- and pyridin-benzotriazol derivative-BRD4 BD1 cocrystal structures revealed that Gln85, Asp88, and Tyr97 interact with the ligand and contribute to the enhancement in ligandability (Fig. 1b, illustrated in aquamarine sticks)^30,31^.

In BD1, there are characteristic conserved water molecules^15^ near the WPF motif (Supplementary Table S1). The water molecules located at conserved positions play critical roles in binding the Kac peptide and maintaining the BD1 structure by forming hydrogen bonds (Supplementary Fig. S3a,b; W1, W2 (2–1, 2–2, 2–3), W3 and W4). Three of them (W1, W3, and W4) existed in highly conserved positions and W2 molecules existed in relatively variable positions in many Kac peptide-bound BD1 structures. In detail, the W1 is the essential water for maintaining the cavity structure by bridging the mainchain carbonyl group of Pro82 and Gln85 and the W4 molecule connects two sidechains of Gln84 and Gln85, respectively (Supplementary Fig. S3). The W2 (including 2–1, 2–2, 2–3) and W3 waters usually generate additional interactions between BD1 and Kac peptides to enhance the Kac peptide-BD1 interactions. Particularly, the W3 involved in multiple interaction between mo-Kac or di-Kac moiety and Pro82. Most of the peptide-bound BD1 structures have these four conserved water molecules and many pan BETi-bound BD1 structures have W1 and W4 molecules. These suggest that disruption of waters (W1 and W4) which involved in shaping of the Kac peptide binding pocket likely unfavorable.

Although these waters are not strictly conserved in ligand-bound BD1 structures, some of them are also found in many inhibitor-bound BD1 structures (Supplementary Fig. S3c). Usually, the W3 water molecules are occupied by inhibitor molecules. Moreover, an inhibitor-bound BD1 structure (PDB code: 7W3D) do not have any four conserved waters. The sulfonamide moiety of *N*2-(1,2,3-benzotriazol-5-yl)-*N*3-(dimethylsulfamoyl)-*N*6-[(2S)-1-methoxypropan-2-yl]pyridine-2,3,6-triamine displaced W2 and other Kac binding pocket shaping waters were not also observed (Supplementary Fig. S3d)^30^.

### Biological activity studies of [1,2,4]triazolo[4,3-*b*]pyridazine derivatives

**Figure 2:**
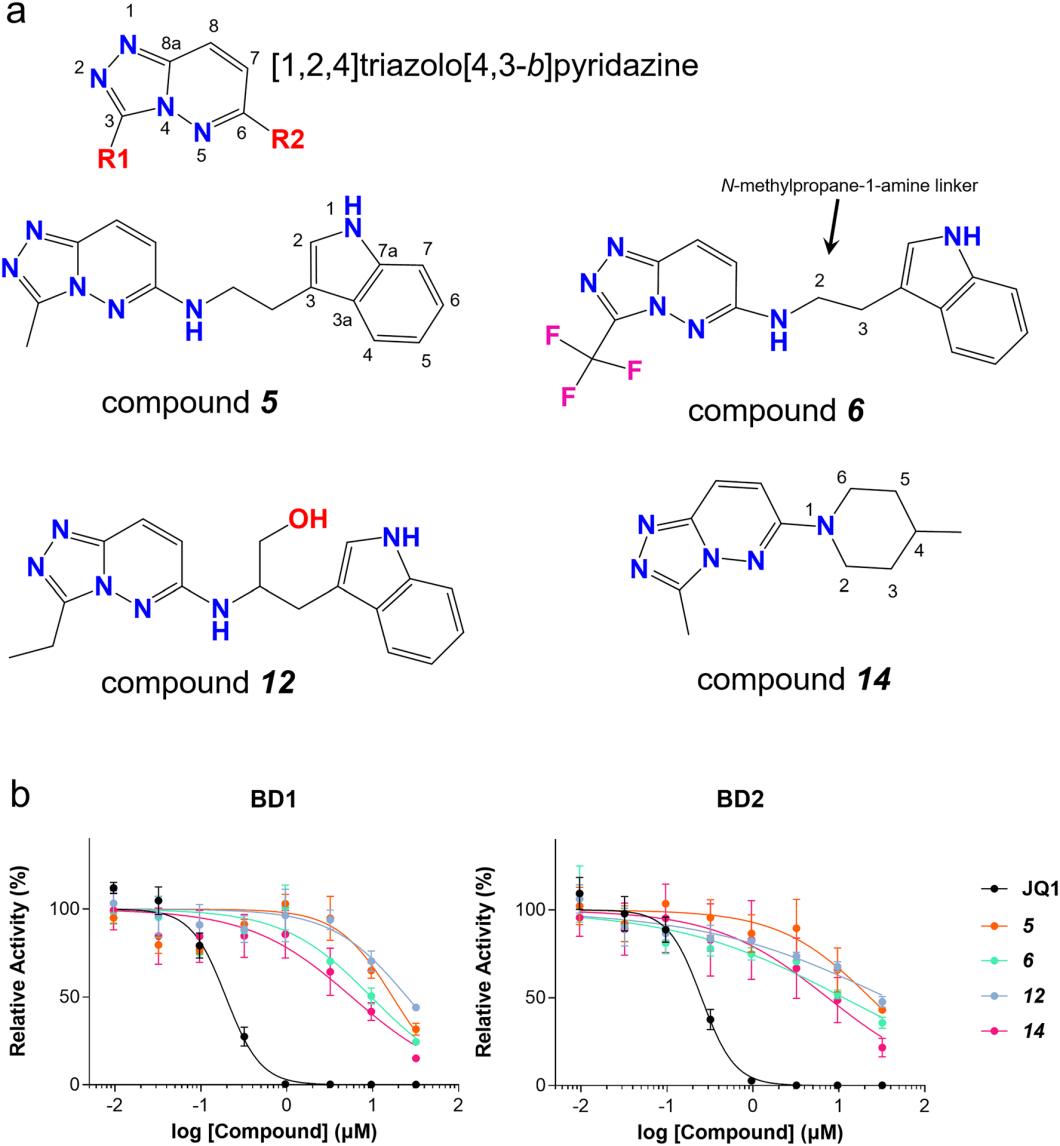
Selected [1,2,4]triazolo[4,3-*b*]pyridazine derivatives for BETi. (**a**) The core scaffold chemical structure for derivative studies and four selected compounds for complex structure determination. (**b**) Representative results for the in vitro inhibition test by AlphaScreen (PerkinElmer, Waltham, MA, USA).

To find the hit molecules for BRD4 bromodomain inhibitor discovery, we performed an in vitro AlphaScreen assay with in-house chemical libraries. The hit molecule and its derivatives were tested to measure the IC_50_ values against BD1 (Fig. 2, Supplementary Table S2). In the first-line screening, we found the compound ***5*** as an initial hit (Supplementary Table S2), which contains [1,2,4]triazolo[4,3-*b*]pyridazine as the chemical core scaffold.

To examine the inhibitory activity according to the chemical structure variations, we replaced the chemical groups at the R1 and R2 sites of the core scaffold and monitored inhibitory activities (Supplementary Table S2). The in vitro tests showed that inhibitory effects were not greatly improved when various indole-based groups were introduced to the R2 site and various chemical groups were introduced at the R1 site (***5***–***12***). In detail, modification of the C5 position of indole with a fluoro group (***7***) or methoxy group (***8***) showed the nearly same blocking efficacy (carbon position numbers are indicated in Fig. 2). In addition, the substitution from methyl to trifluoromethyl at R1 did not much improved the IC_50_ value (***6***). Other modifications were conducted at the C2 and C3 positions in *N*-methylpropan-1-amine, a linker between the core scaffold and indole. The trifluoromethyl moiety at R1 and aryl addition at C3 in linker (***9***) increased the IC_50_ value by only twofold compared to that of ***6***, which suggests that the addition of an aryl moiety on the ethylene linker showed no positive effect on BD1 binding. Furthermore, changes from methyl to ethyl at R1 and methanol addition at the C2 position in the *N*-methylpropan-1-amine linker (***12***) showed decreased blocking efficiency compared to that of ***5***. The inhibitory efficacy was worse (no inhibition) with the bulky ortho fluorobenzyl substitution at R1 and the formation of a piperidine ring between indole and [1,2,4]triazolo[4,3-*b*]pyridazine (***11***). Methylcyclopropane substitution at R1 and chloro- and fluoro- ortho-substitution at the C5 and C6 positions of indole showed no inhibitory efficiency in the measured range (***10***).

A series of chemical optimization trials by changing the R2 with piperidine derivatives (***13***–***27***) provided very diverse results. While substitution of only the piperidine group at R1 (***13***) showed no blocking activities, but surprisingly, introducing 4-methylpiperidine (***14***) restored inhibitory efficacy. Next, we further examined an additional thirteen piperidine-based derivatives (***15***–***27***). *N*,*N*-dimethylmethane sulfonamide substitution at R1 with compound ***14*** (***15***) and oxygen and sulfur substitution at the C4 position of piperidine (***16***, ***17***, ***18***) showed no inhibitory efficacy. Only two derivatives (***19***, ***21***) exhibited inhibitory activities for spiro ring compound substitution at the C4 position of piperidine (***19***–***23***). Additionally, R1 substitution with hydrophobic chains and cyclic rings as well as R2 substitution at the C4 position of piperidine showed no inhibitory activities (***24***–***27***). When linear chains were substituted at R2 (***28***–***34***), only an ethylpropanoate-containing derivative (***33***) showed inhibitory activity, suggesting difficulties in BD1 binding with these derivatives. Substitution of the benzyl group instead of the indole group (***35***–***37***) resulted in similar or slightly improved activities. However, benzyl groups with longer amide-based linkers (***38***–***40***) showed no inhibitory activities.

We also tested inhibitory activities with four selected hit compounds (compounds ***5***, ***6***, ***12***, and ***14***) for BD2 and found that these compounds also have inhibitory activities for BD2, suggesting the pan BETis (Fig. 2b; Table 1).

**Table 1:** IC_50_ values of selected [1,2,4]triazolo[4,3-*b*]pyridazine derivatives against BD1 and BD2.

Compounds	JQ1^a^	*5*	*6*	*12*	*14*
IC_50_ (BD1)^b^	195.1 (± 14.2) nM	17.1 (± 3.1) μM	9.6 (± 2.0) μM	25.2 (± 4.6) μM	5.7 (± 1.4) μM
IC_50_ (BD2)^b^	251.4 (± 16.0) nM	23.2 (± 5.2) μM	11.3 (± 3.2) μM	36.5 (± 12.3) μM	7.4 (± 2.2) μM

^a^Positive control.

^b^N = 3, standard deviations are represented in parentheses.

### Structural studies of BD1:[1,2,4]triazolo[4,3-*b*]pyridazine derivatives

**Figure 3:**
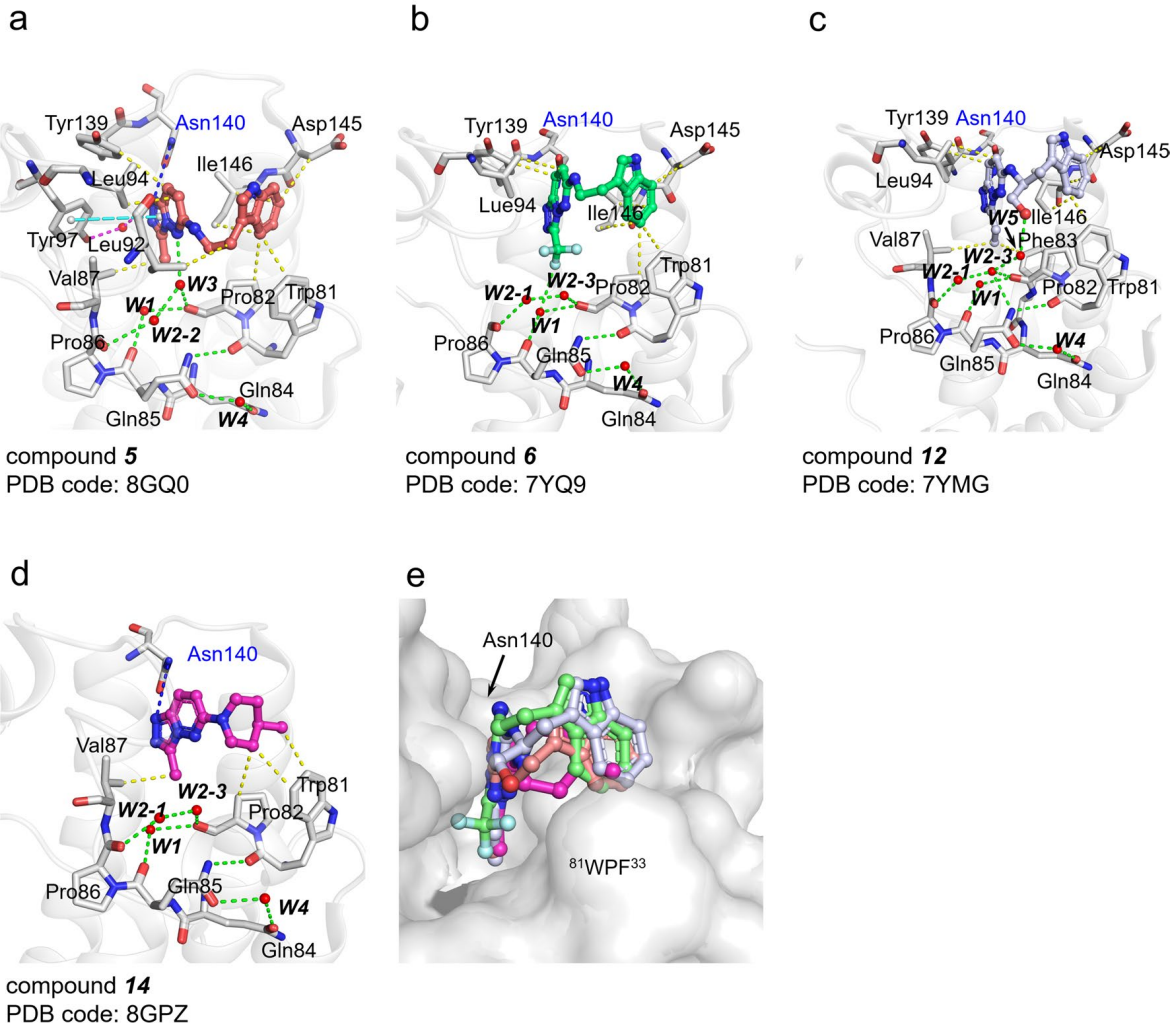
Cocrystal structure of each compound in BRD4 BD1. (**a**–**d**) The compound ***5***,*** 6***, ***12***, ***14***-bound BD1 structures. Each interaction is illustrated by dashed lines (hydrogen bond (blue dash), π–π stacking interaction (cyan dash), hydrophobic interaction (yellow dash), conserved water molecule-mediated interactions (green dash)). (**e**) Superposition of four ligand-bound BD1 structures. The protein–ligand interactions were analyzed and quantified using PLIP servery^32,33^. The figures were drawn using the program PyMOL version 2.0 (Schrödinger, New York, NY, USA).

To explain the rationales of the inhibitory activities of the derivatives, we tried to determine the crystal structure of BD1 in complex with inhibitors. We successfully obtained four crystal structures of BD1 in complex with [1,2,4]triazolo[4,3-*b*]pyridazine derivatives (compounds ***5***, ***6***, ***12***, and ***14***; Figs. 2 and 3). All the crystal structures were high-resolution structures (1.4 − 1.53 Å; Supplementary Table S3, Supplementary Fig. S4).

Most of the published Kac mimetic BRD4 BD1 inhibitor-bound structures showed that their interactions use conserved key interacting residues and water molecules. Our [1,2,4]triazolo[4,3-*b*]pyridazine derivative structures showed similar binding modes (Fig. 3). The overall orientations of [1,2,4]triazolo[4,3-*b*]pyridazine are nearly the same in all four compounds. The conserved WPF motif interacts with chemical groups at the R1 site and indole (or methylpiperidine) groups at the R2 site of the hit (Fig. 3).

Among many interacting key residues that identified by PLIP server analyses, each derivative utilizes approximately five to ten residues for interactions. In detail, chemical groups at the C3 position of the core scaffold methyl (***5***, ***14***), trifluoromethyl (***6***), and ethyl (***12***) toward the WPF motif in the Kac binding pocket and indole or methylpiperidine ring are exposed to the solvent region but also toward the WPF motif. The N1 nitrogen atom of the triazole of the core scaffold forms hydrogen bonds with Asn140. Two hydrophobic interactions between the core scaffold and Leu94 and the core scaffold and Tyr139 are common features in ***5***, ***6***, and ***12***. These hydrophobic interactions are not found in ***14*** (Fig. 3d). In addition, the hydrophilic trifluoromethyl moiety in ***6*** lost its hydrophobic interaction with Val87, while others maintained this interaction. The triazole moiety of*** 5*** was pulled to Asn140, and it generated π-stacking interactions with Tyr97 as well as water-mediated hydrogen bond interactions (Fig. 3a). A superposition of all four structures showed slight positional movement of the core scaffold in the ***5-***, ***6-***, ***12-***, and ***14-***bound BD1 structures (Fig. 3e). There is no large conformational change in BRD4 BD1 in the [1,2,4]triazolo[4,3-*b*]-BRD4 BD1 complex structures, and ligand recognition is likely governed by conformational selection rather than induced fit^34^.

Another common feature of the compound ***5-***, ***6-***, ***12-***BD1 complex is that the indole group contacts Trp81, Asp145, and Ile146 through hydrophobic interactions (Fig. 3a–c). On the other hand, there are also differences between the three complex structures for adapting conformational selection; the indole group of ***12*** is more bent and does not form hydrophobic interactions with Pro82. The compound ***14*** contains a piperidine group at the R2 position of the scaffold instead of indole, which does not interact with Asp145 and Ile146 to form hydrophobic interactions but still interacts with Trp81 and Pro82 (Fig. 3d).

The conserved water molecules in the ligand binding site play different roles according to inhibitor type. The W1 molecule in the ***6-***BD1 complex contributed to an additional hydrogen bond with the trifluoromethyl moiety (Fig. 3b). The hydroxyl group in ***12*** adopted an additional water molecule (W2-1, W2-3, and W5)-mediated interaction for ligand binding (Fig. 3c). The compound ***14*** showed a retained conserved water with the WPF motif, but these water molecules did not participate in water-mediated ligand binding (Fig. 3d). Only **5** contains W3, similar to Kac peptide, and it interacts with N5 of the imine group at [1,2,4]triazolo[4,3-*b*]pyridazine core scaffold, forming a water-mediated interaction (Fig. 3a). The ***12-***BD1 complex provides insight for drug design because the ethyl moiety of R1 was bent deep inside the cavity due to its hydrophobicity (Fig. 3c). It could also be inferred from our structures that hydrophobic R1 substitutions, such as methylcyclopropane, methylcyclobutane, and isobutane (***10***, ***24***–***27***), may not be capable of displacing W1 and W2 waters to bind and inhibit BD1 (Fig. 3).

**Figure 4:**
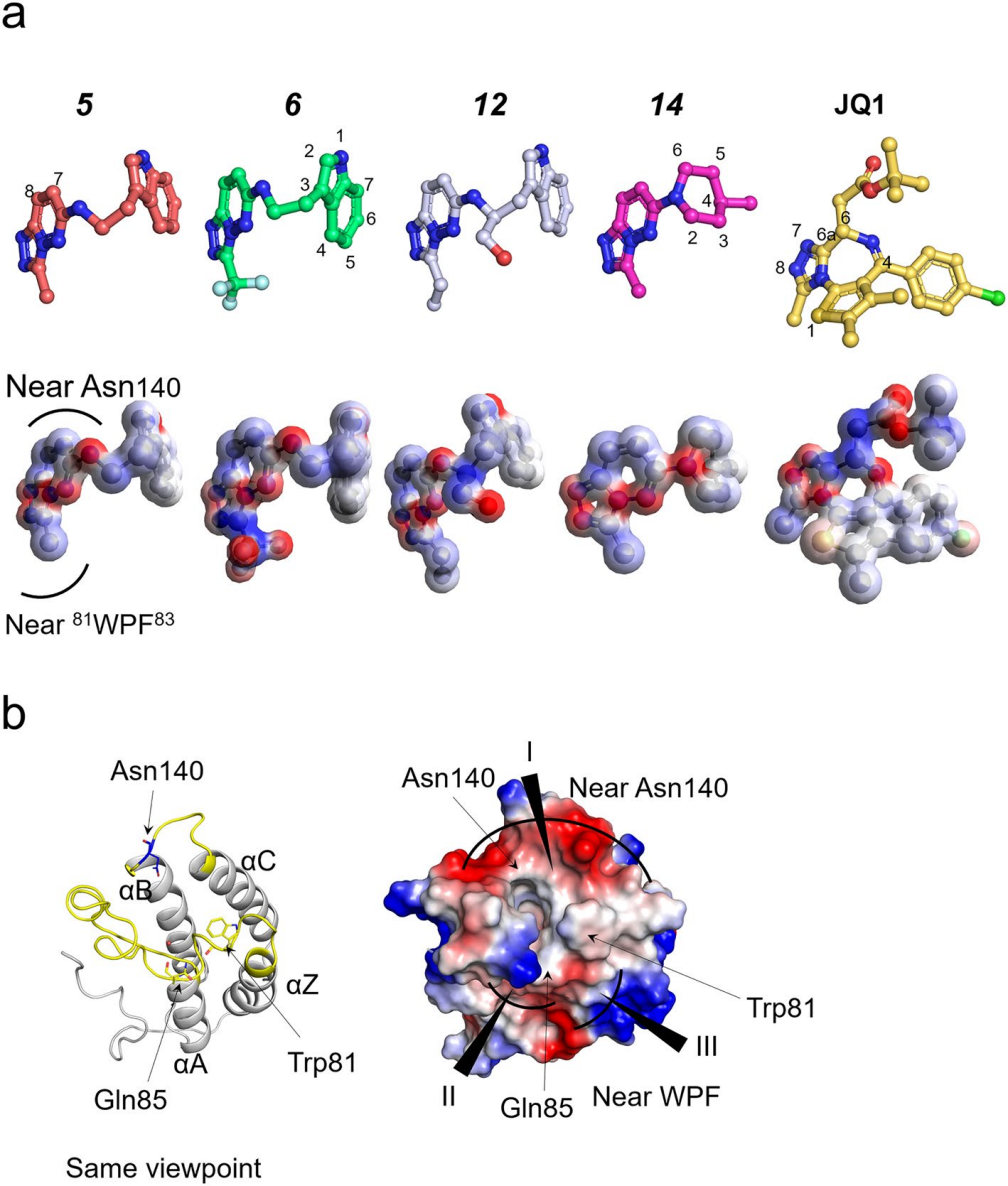
Illustration of the electrostatic surface of each compound and BRD4 BD1. (**a**) The electrostatic surface of [1,2,4]triazolo[4,3-*b*]pyridazine derivatives. (**b**) The electrostatic potential surface of BRD4 BD1. The figures were drawn using the program Avogadro version 1.2.0 (http://avogadro.cc/) PyMOL version 2.0 (Schrödinger, New York, NY, USA).

Bromodomains are structurally conserved domains in a broad range of proteins, but they have unique and distinct ligands. It has been suggested that a variety of bromodomain-containing proteins exhibit sequence selectivity for Kac peptides of histone and nonhistone proteins due to the electrostatic properties of the cavity^1,8^. Electrostatic complementarity assessment between protein and ligand surfaces is an important consideration for drug discovery^35^. In this manner, we created the electrostatic potential (ESP) surface of each compound, including the potent pan BETi JQ1, with the ESP surface visualization suite in Avogadro^36^ (Fig. 4a). Interestingly, the charged regions of the compounds and the surface of the BD1 ligand binding cavity were electrostatically complementary (Fig. 4). Because diazene N1 and N2 were relatively negatively charged, the C7 and C8 positions of [1,2,4]triazolo[4,3-*b*]pyridazine showed a relatively positive charge and were positioned near Asn140 (Fig. 4b, region I), which is the negatively charged surface. The C4 and C5 positions in indole and the C2 and C3 positions in piperidine were electrically neutral, and they interacted with Trp81, which is an electrically neutral residue (Fig. 4b, region III). It is an exception that the fluorine atoms of the trifluoromethyl moiety (***6***), which are highly negative, were close to electrically neutral region II. However, the fluorine atom of ***6*** did not directly contact neutral region II but formed water-mediated interactions (Fig. 3). The compound ***14***, the smallest compound in this study, showed that the surfaces of the compound and binding position of BD1 were electrostatically complementary as well. As shown in Fig. 4b, JQ1 represents a strongly electrical complement surface with BRD4 BD1. The C6 and C6a positions of thienotriazolodiazepines are strongly positively charged and interact with region I. In addition, the dimethylthiophene of JQ1 is neutral and faces region II. Similar to our structure, it interacted with region II through water-mediated interactions. However, the W3 position was occupied by a dimethylthiophene group, and the sulfur atom formed a hydrogen bond with W2 (Supplementary Fig. S3c, magenta dashes)^37,38^.

## Conclusions

Overall, we found a series of [1,2,4]triazolo[4,3-*b*]pyridazine derivatives as pan BETi scaffolds. As a structural activity relationship study, we studied four groups of chemical derivatives in R2, which were indole-, aryl-, aliphatic-, and piperidine-based moieties. Additionally, a small change in R1 was also conducted at the same time. The study showed that a series of [1,2,4]triazolo[4,3-*b*]pyridazine derivatives showed micromolar IC_50_ values in BRD4 BD1 inhibition. The actual binding characteristics of the four compounds could be explained by X-ray cocrystal structural studies. All four compound-bound structures showed that BD1 residues, which are already known to be important for ligand binding, also participated in the four ligand interactions. In addition, the conserved water molecules, shown in many previous structure reports, also conserved our inhibitor-bound BD1 structures, contributing to ligand binding. Finally, the electrostatic potential surface of each inhibitor and BRD4 BD1 were studied, and ligand interactions could be explained. Through these studies, we proposed [1,2,4]triazolo[4,3-*b*]pyridazine derivatives as starting points for the structure-based discovery of BETis.

## Methods

### BRD4 BD1 expression and purification

BRD4 bromodomain 1 (BD1; 44 − 168) protein expression and purification were performed according to a previous method. The BRD4 BD1 plasmid, subcloned into a modified pET28b vector (TEV cleavage site inserted at N-terminal; BamHI, XhoI), was transformed into BL21(DE3) and cultured with Terrific Broth. After 0.1 mM isopropyl-D-thiogalactoside induction when the OD_600_ reached approximately 0.8, the cells were incubated with vigorous shaking at 18 °C overnight. After harvest, the cells were resuspended, and recombinant BRD4 BD1 was purified with Ni–NTA resin. To cleave the His_6x_ tag, 2.5 mg of TEV protease was added to 100 mg of purified BRD4 BD1. Further purification was performed with Ni–NTA resin to collect the cleaved BRD4 BD1 with the flowthrough fraction, and size-exclusive chromatography was performed to increase the purity of the recombinant protein. BRD4 BD1 was concentrated to 12 mg/mL, which was measured by nanodrop, converted with the calculated extinction coefficient, and stored at −80 °C until crystallization.

### AlphaScreen enzyme assay

The AlphaScreen assay was described previously and performed according to the manufacturer’s protocol (PerkinElmer, USA). The reaction buffer was 50 mM HEPES pH 7.4, 100 mM NaCl, 0.1% BSA, and 0.05% CHAPS. The whole reaction and screening were performed using OptiPlateTM-384 (PerkinElmer, Waltham, MA, USA).

The compounds, acetylated peptide [SGRGK(Ac)GGK(Ac)GLGK(Ac)GGAK(Ac)RHRK-biotin] and purified bromodomain protein were added to the OptiPlateTM-384 plate. Then, streptavidin-coated donor beads and anti-GST AlphaScreening acceptor beads were added. The incubation condition was 25 °C for 1 h using a Thermomixer C (Eppendorf, USA). The blocking efficacy (IC_50_) values were obtained by 8-point titration (from 0.0096 to 32 μM). The IC_50_ values of inhibitors were evaluated with GraphPad Prism 7 using normalized and inhibition analysis suites.

### Chemistry

**Scheme 1:**
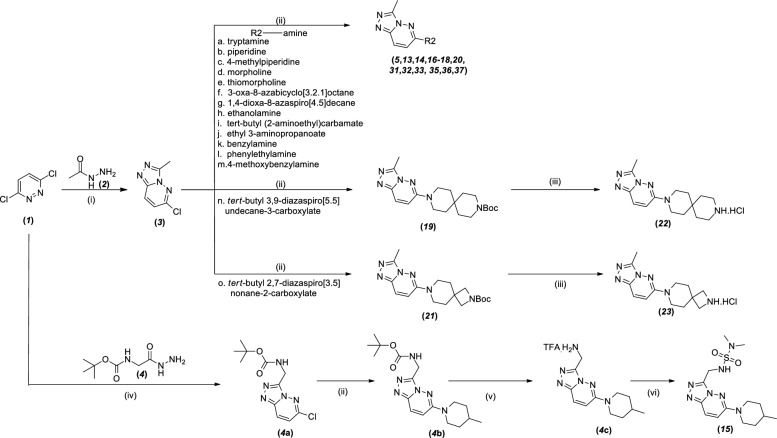
Synthetic route of compounds ***5, 13***–***23, 31***–***33*** and ***35***–***37***^a^. ^a^Reagents and conditions: (i) anhydrous n-BuOH, 120 °C, 16 h; (ii) KI, conc. HCl, ethanol, 80 °C, 24 h/72 h; (iii) 4 M HCl in dioxane: (iv) *p*-TSA, anhydrous ethanol, 80 °C, 14 h; (v) TFA, DCM, 1 h: (vi) *N*,*N*-dimethylsulfamoyl chloride, pyridine, 0 °C to RT, 2 h.

Most of the compounds were synthesized by the following procedure in Scheme 1 (detailed description are in Chemistry experimental section in Supplementary materials S1). Unless otherwise stated, all reactions were performed under an inert (N_2_) atmosphere. Reagents and solvents were of reagent grade and purchased from Sigma‒Aldrich, Alfa Aesar, and TCI Tokyo. Flash column chromatography was performed using silica gel 60 (230–400 mesh, Merck) with the indicated solvents. Thin-layer chromatography was performed using 0.25 mm silica gel plates. Proton nuclear magnetic resonance (^1^H NMR) spectra were recorded on a BRUKER ultrashield 300 MHz NMR spectrometer at 25 °C. Chemical shifts are reported in parts per million (ppm). ^1^H NMR data are reported as follows: chemical shift (δ ppm) (multiplicity, integration, coupling constant [Hz]). Multiplicities are reported as follows: s = singlet, d = doublet, t = triplet, q = quartet, and m = multiplet. The residual solvent peak was used as an internal reference. The mass spectra were obtained using Acuity™ waters A06UPD9BM and Agilent Technologies SG12109048. Prior to biological testing, the final compounds were confirmed to be > 95% pure by ultra-performance liquid chromatography (UPLC) using a Waters ACQUITY H-class system fitted with a C18 reversed-phase column (ACQUITY UPLC BEH C18: 2.1 mm × 50 mm, Part no. 186002350) according to the following conditions: (A) H_2_O + 0.1% formic acid, (B) CH_3_CN + 0.1% formic acid, (C) methanol (MeOH) + 0.1% formic acid; (Ι) a gradient of 95% A to 95% B over 5 min, (II) a gradient of 95% A to 95% C over 5 min.

Scheme 1 shows the chemical synthesis of compounds (***5***, ***13***–***23***, ***31***–***33*** and ***35***–***37***), starting with compound ***1*** and acetohydrazide (***2***), which were obtained commercially and reacted together in anhydrous n-butanol to generate cyclized compound ***3***. The compound ***3*** was further reacted with amines (**a**-**o**) using catalytic amounts of KI and conc. HCl (concentrated HCl, 12N HCl) together in ethanol inside a sealed tube at 80 °C for 24 and/or 72 h to obtain the corresponding compounds (***5***, ***13***, ***14***, ***16***–***21***, ***31***–***33***, ***35***–***37***). Compounds ***22*** and ***23*** were obtained by deprotecting the Boc group using 4 M HCl in dioxane.

**Scheme 2:**
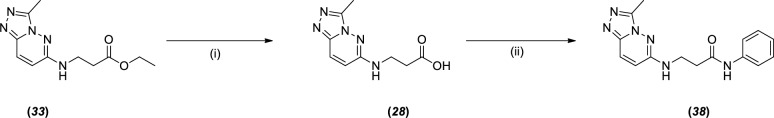
Synthetic route of compounds ***28*** and ***38***^a^. ^a^Reagents and conditions: (i) LiOH. H_2_O, THF, H_2_O, 2 h, RT; (ii) Aniline, HATU, TEA, acetonitrile 3 h, RT.

Intermediate ***4*a** was synthesized by reacting ***1*** with commercially available tert-butyl (2-hydrazineyl-2-oxoethyl)carbamate by employing condition (iv) of Scheme 1. The obtained ***4a*** was subjected to nucleophilic substitution by 4-methylpiperidine using condition (ii) of Scheme 1 to produce intermediate ***4*b,** which was further treated with TFA and DCM to remove the Boc group and generate intermediate ***4*c**. Intermediate ***4*c** was reacted with *N*,*N*-dimethylsulfamoyl chloride and base pyridine to produce compound ***15***. Throughout the synthesis of compounds shown in the scheme, they were obtained in reasonable yields unless otherwise stated (Scheme 2).

**Scheme 3:**
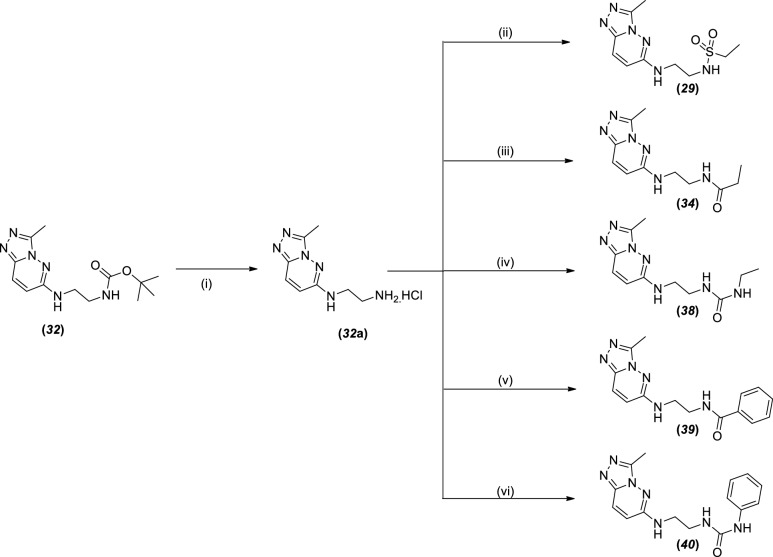
Synthetic route of compounds ***29****, ****34*** and ***38***–***40***^a^. ^a^Reagent and conditions: (i) 4 M HCl in dioxane, 1 h, RT; (ii) ethanesulfonyl chloride, DIPEA, anhydrous ethanol, 0 °C to RT, 2 h; (iii) propanoic acid, HATU, TEA, anhydrous *N*,*N*-dimethylformamide, 2 h, RT; (iv) ethyl isocyanate, DIPEA, acetonitrile, 5 h, RT; (v) benzoyl chloride, pyridine, 0 °C to RT, 4 h; (vi) phenyl isocyanate, DIPEA, acetonitrile, RT, 5 h.

Compound ***33*** was obtained as described in Scheme 1 and then hydrolyzed by treatment with lithium hydroxide monohydrate to generate compound ***28***. This compound was reacted with the peptide coupling agent HATU, base TEA and aniline to generate compound ***38*** with a lower yield of 17% (Scheme 3).

Compound ***32*** from Scheme 1 was treated with 4 M HCl in dioxane to achieve intermediate ***32*a** as an amine-monohydrochloride salt, which was then further subjected to the synthesis of five different compounds (***29***, ***34***, ***38***–***40***). Compound ***29*** was achieved by reaction between intermediate ***32*a** and ethanesulfonyl chloride in solvent ethanol and DIPEA as base; compound ***34*** was obtained by reaction among intermediate ***32*a**, propionic acid and peptide coupling reagent HATU. Compounds ***38*** and ***40*** were obtained in a similar manner by reaction of intermediate ***32*a** with the corresponding isocyanate reagents. Intermediate ***32*a,** when reacted with benzoyl chloride and base pyridine, produced compound ***39***. The overall isolated yield was lower than that of compounds ***38*** and ***40***.

### Chemicals

Some chemical derivatives of compound ***5*** were searched using online chemical similarity search tools (https://mcule.com/search/) and purchased for further studies. Before use, all compounds were dissolved in 100% dimethyl sulfoxide (DMSO) as stock solutions of 100 mM. The supplier and catalog number are described below.

*N*-[2-(1*H*-indol-3-yl)ethyl)-3-(trifluoromethyl)-[1,2,4]triazolo[4,3*-b*]pyridazin-6-amine (Vitas-M, STK651245) (***6***), *N*-[2-(5-fluoro-1*H*-indol-3-yl)ethyl]-3-methyl-[1,2,4]triazolo[4,3*-b*]pyridazin-6-amine (Enamine, Z1220635364) (***7***), *N*-[2-(5-methoxy-1*H*-indol-3-yl)ethyl)-3-methyl-[1,2,4]triazolo[4,3*-b*]pyridazin-6-amine (Enamine, Z1192171101) (***8***), *N*-[2-(1*H*-indol-3-yl)-2-phenylethyl]-3-(trifluoromethyl)-[1,2,4]triazolo[4,3-*b*]pyridazin-6-amine (Enamine, Z219181640) (***9***), *N*-[2-(5-chloro-6-fluoro-1*H*-indol-3-yl)ethyl]-3-cyclopropyl-[1,2,4]triazolo[4,3-*b*]pyridazin-6-amine (Enamine, Z2701558508) (***10***), 6-Fluoro-2-[3-(2-fluorophenyl)-[1,2,4]triazolo[4,3-*b*]pyridazin-6-yl]-1,3,4,9-tetrahydropyrido[3,4*-b*]indole (Enamine, Z4500949681) (***11***), 2-[(3-ethyl-[1,2,4]triazolo[4,3-*b*]pyridazin-6-yl)amino]-3-(1*H*-indol-3-yl)propan-1-ol (Z1272770102) (***12***), 1-(3-Isopropyl-[1,2,4]triazolo[4,3-*b*]pyridazin-6-yl)piperidine-4-carboxylic acid (Vitas M, STK719914) (***24***), *N*-(3-methylbutyl)-1-[3-(propan-2-yl)[1,2,4]triazolo[4,3-*b*]pyridazin-6-yl]piperidine-4-carboxamide (Vitas M, STK635934) (***25***), *N*-cyclopropyl-1-[3-(propan-2-yl)[1,2,4]triazolo[4,3-*b*]pyridazin-6-yl]piperidine-4-carboxamide (Vitas M, STK6497959) (***26***), 3-cyclobutyl-6-(4-isopropylpiperazino)[1,2,4]triazolo[4,3-*b*]pyridazine (ChemDiv, Z606-3990) (***27***).

### Complex structural study of BRD4 BD1 and selected compounds

All crystals were obtained by the hanging-drop vapor-diffusion method at 14 °C, following previous protocols^30,31^. Before crystallization, the BRD4 BD1 protein stock was thawed slowly on ice, and the protein was diluted tenfold (1.2 mg/mL) with storage buffer (10 mM HEPES-OH pH 7.5, 500 mM NaCl, 5% (v/v) glycerol, and 10 mM dithiothreitol). In addition, compounds were mixed with a tenfold molar ratio compared to the protein stock and incubated at 4 °C with mild rotation overnight. The next day, BRD4 BD1 and compound solutions were concentrated tenfold, and the stocks were used for vapor diffusion crystallization by hanging drop. Each protein-inhibitor mixture and reservoir solution were mixed at a 1:1 ratio, and the formulation was sodium formate (ranging from 5 to 6 M) and glycerol (ranging from 2 to 12%). Crystals can be observed within 3 days and were grown over 10 days for full growth.

The diffraction dataset was collected using either the Pohang Light Source (PLS) Beamline 11C station with a Dectris Pilatus 6 M detector or the PLS Beamline 7A station with an ADSC Quantum 270 detector (Pohang, Korea). The reflected raw dataset was scaled by HKL2000^39^. Molecular replacement solutions were processed by the PHASER suite in the PHENIX program suite, and the BRD4 BD1 structure (PDB code: 6KEK) was used as a search model^31,40,41^. Real space refinement and refinement were processed using the programs COOT and PHENIX, with repeated cycles^42^. Coordinates and restraint files of compounds were generated by MAESTRO (Schrödinger, New York, NY, USA) and the eLBOW suite in the PHENIX program^43^. Electron density maps for each inhibitor are shown in Supplementary Fig. S4.

### Electrostatic potential surface creation of compounds

The electrostatic potential surface creation was conducted by the “create surfaces” suit. The electrostatic potential surface creation was conducted by Avogadro version 1.2.0 (http://avogadro.cc/)^36^. The force field of each compound was changed as a universal force field (UFF), and then surface creation was processed. The chemical structure was extracted from each deposited PDB data to preserve the stereochemistry of the conformational selection.

## Supplementary Information

Supplementary Information.

## Data Availability

All crystallographic coordinates and structure factors were deposited in the PDB under the accession codes 8GQ0 (compound ***5***), 7YQ9 (compound ***6***), 7YMG (compound ***12***), and 8GPZ (compound ***14***).
