# Supplementary material for: Crystal structure of [1,2,4] triazolo[4,3- b ]pyridazine derivatives as BRD4 bromodomain inhibitors and structure–activity relationship study
Source: Sci Rep. 2023 Jul 4;13:10805. doi: 10.1038/s41598-023-37527-w (PMC10319850; doi:10.1038/s41598-023-37527-w)
Supplement: Supplementary file 1 — Supplementary Information. [file 41598_2023_37527_MOESM1_ESM.pdf]

Supplementary information

Crystal structure of [1,2,4]triazolo[4,3-*b*]pyridazine derivatives as BRD4 bromodomain inhibitors and structure-activity relationship study

Jung-Hoon Kim[1,2,6], Navin Pandit[3,6], Miyoun Yoo[4,6], Tae Hyun Park[5,6], Ji U Choi[3,4], Chi Hoon Park[3,4,*], Kwan-Young Jung[3,4,*], Byung Il Lee[1,2,*]

[1]Research Institute, National Cancer Center, Goyang, Gyeonggi 10408, Republic of Korea

[2]Department of Cancer Biomedical Science, National Cancer Center Graduate School of Cancer Science and Policy, Goyang, Gyeonggi 10408, Republic of Korea

[3]Department of Medicinal Chemistry and Pharmacology, University of Science & Technology, Daejeon 34113, Republic of Korea

[4]Therapeutics & Biotechnology Division, Korea Research Institute of Chemical Technology, Daejeon 34114, Republic of Korea

[5]Department of Anesthesiology, Weill Cornell Medical College, New York, NY 10065, USA

[6]These authors contributed equally

*Corresponding authors: Chi Hoon Park (chpark@krict.re.kr), Kwan-Young Jung (krjeong@krict.re.kr), Byung Il Lee (bilee@ncc.re.kr)

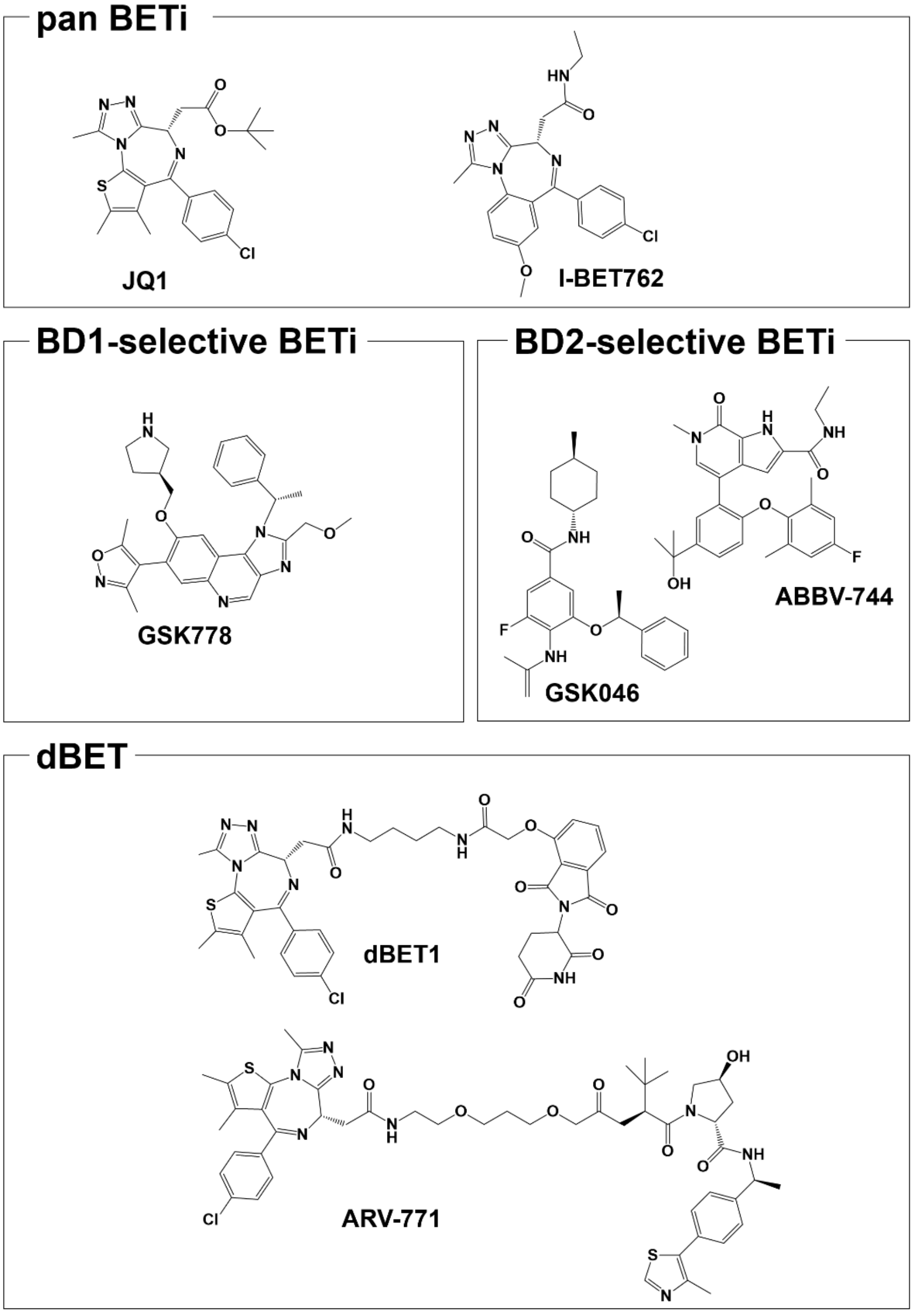

Supplementary Figure S1. Examples of BET BD inhibitors. JQ1 and I-BET762 are representative pan BETis that target BD1 and BD2. GSK778 is a BD1-selective BETi. GSK046 and ABBV-774 are BD2-selective BETis. The dBET compounds consist of the following moieties: an E3-ligase anchoring moiety, a linker, and a BET targeting moiety. The representative dBET compounds (dBET1 and ARV-771) utilize JQ1 as the BET-targeting moiety.

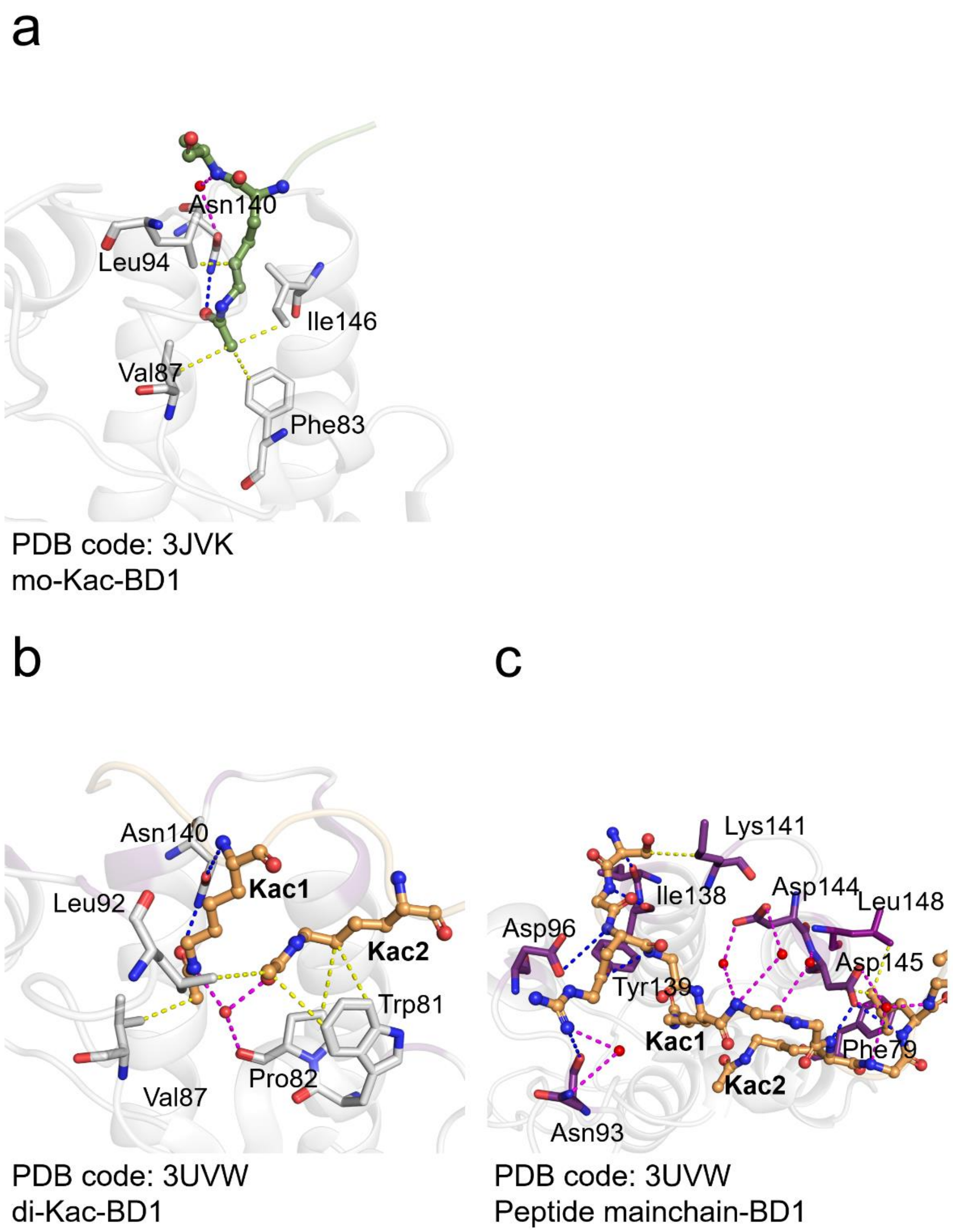
a
Asn140
Leu94
Ile146
Val87
Phe83
PDB code: 3JVK
mo-Kac-BD1
b
Asn140
Leu92
Kac1
Kac2
Trp81
Pro82
Val87
PDB code: 3UVW
di-Kac-BD1
c
Lys141
Ile138
Asp144
Leu148
Asp96
Asp145
Tyr139
Kac1
Phe79
Kac2
Asn93
PDB code: 3UVW
Peptide mainchain-BD1

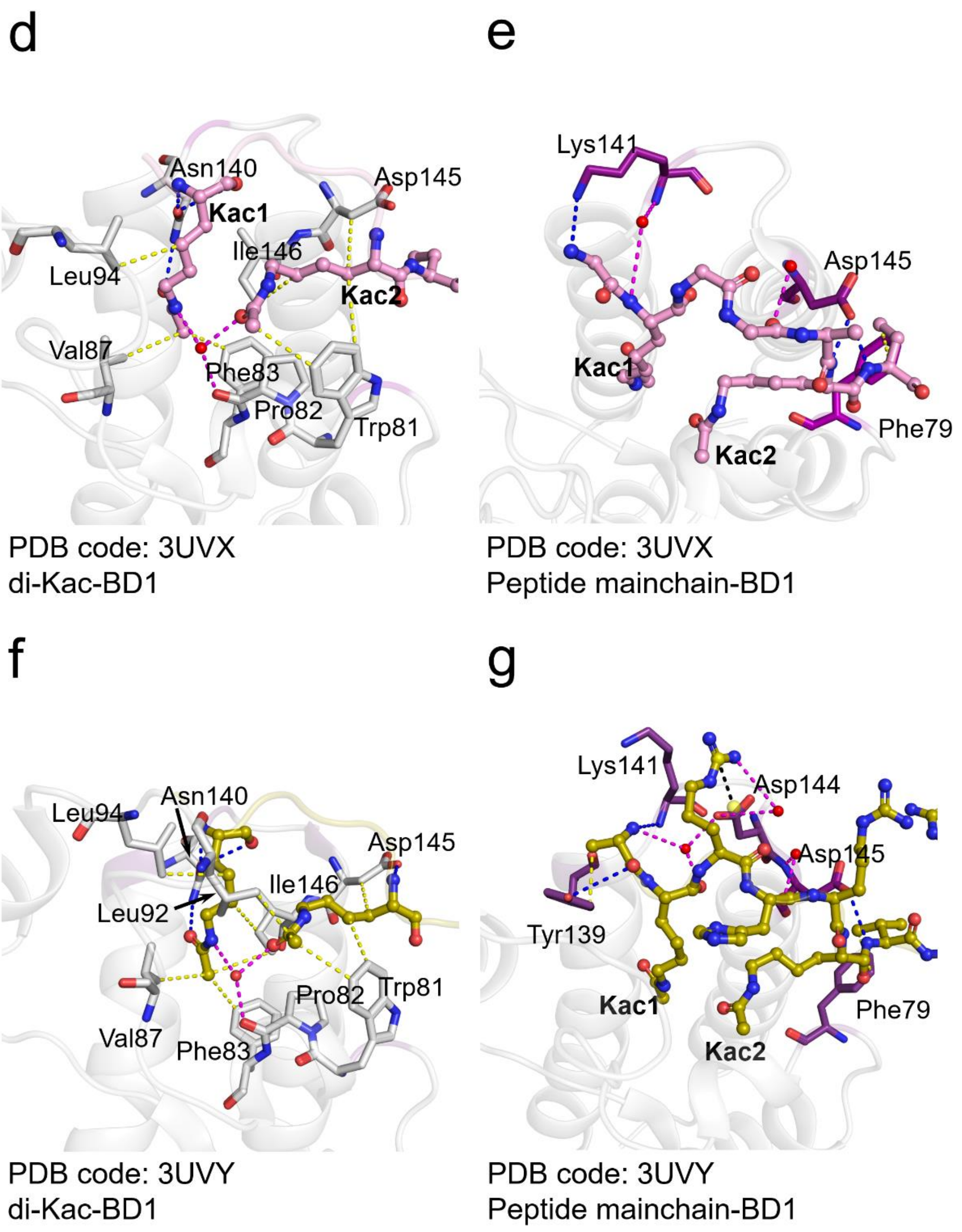

Supplementary Figure S2. Kac-bound BRD4 BD1 structures. (a) A detailed view of mo-Kac–BRD4 BD1 interactions. (b-g) Detailed views of the interactions between the di-Kac peptide and BRD4 BD1. Interactions between BD1 and the sidechains of Kacs (b, d, f) and BD1 and the main chains of Kac peptides (c, e, g) are presented. Hydrogen bonds, hydrophobic interactions, and water-mediated interactions are drawn in blue dashes, yellow dashes, and magenta dashes, respectively. The figures were drawn using the program PyMOL version 2.0 (Schrödinger, New York, NY, USA).

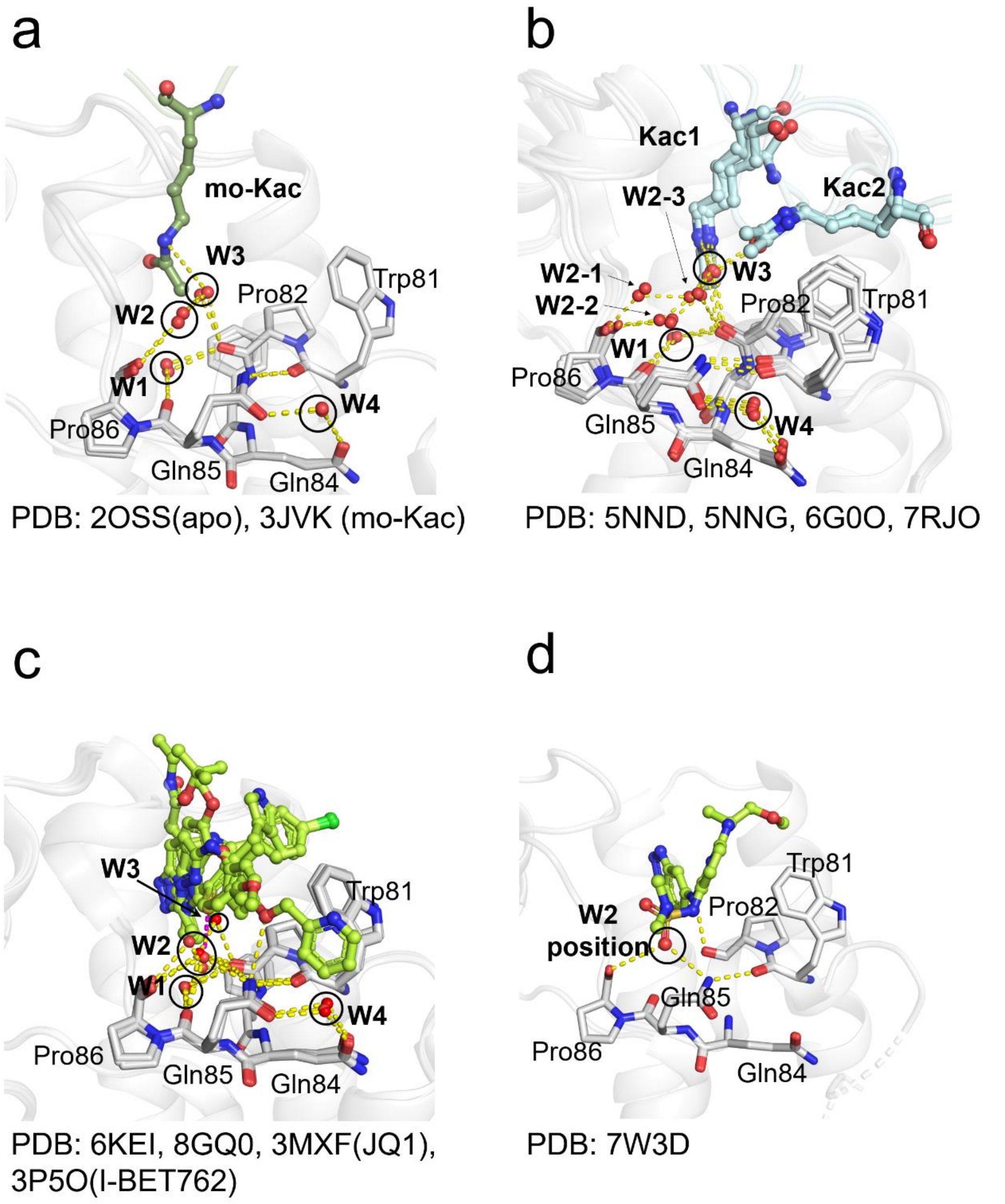

Supplementary Figure S3. Conserved water molecules in BRD4 BD1 near the WPF. (a) Superposition of apo-BRD4 BD1 (PDB code: 2OSS) and mo-Kac-bound BRD4 BD1 (PDB code: 3JVK (H3Kac14)), (b) Superposition of di-Kac-bound BRD4 BD1 (PDB code: 5NND (H3K9ac/K14ac), 5NNG (SRPK1 peptide K585ac), 6G0O (ATRX peptide K1030ac/K1033ac), 7RJO (hnRNPK)). (c) Superposition of many inhibitor-bound BRD4 BD1 cocrystal structures with waters near WPF (PDB codes: 6KEI, 8GQ0, 3MXF(JQ1), 3P5O(I-BET762)). (d) Pyridin-benzotriazol derivative-BRD4 BD1 cocrystal structures (PDB code: 7W3D) showing the loss of conserved water. The figures were drawn using the program PyMOL version 2.0 (Schrödinger, New York, NY, USA).

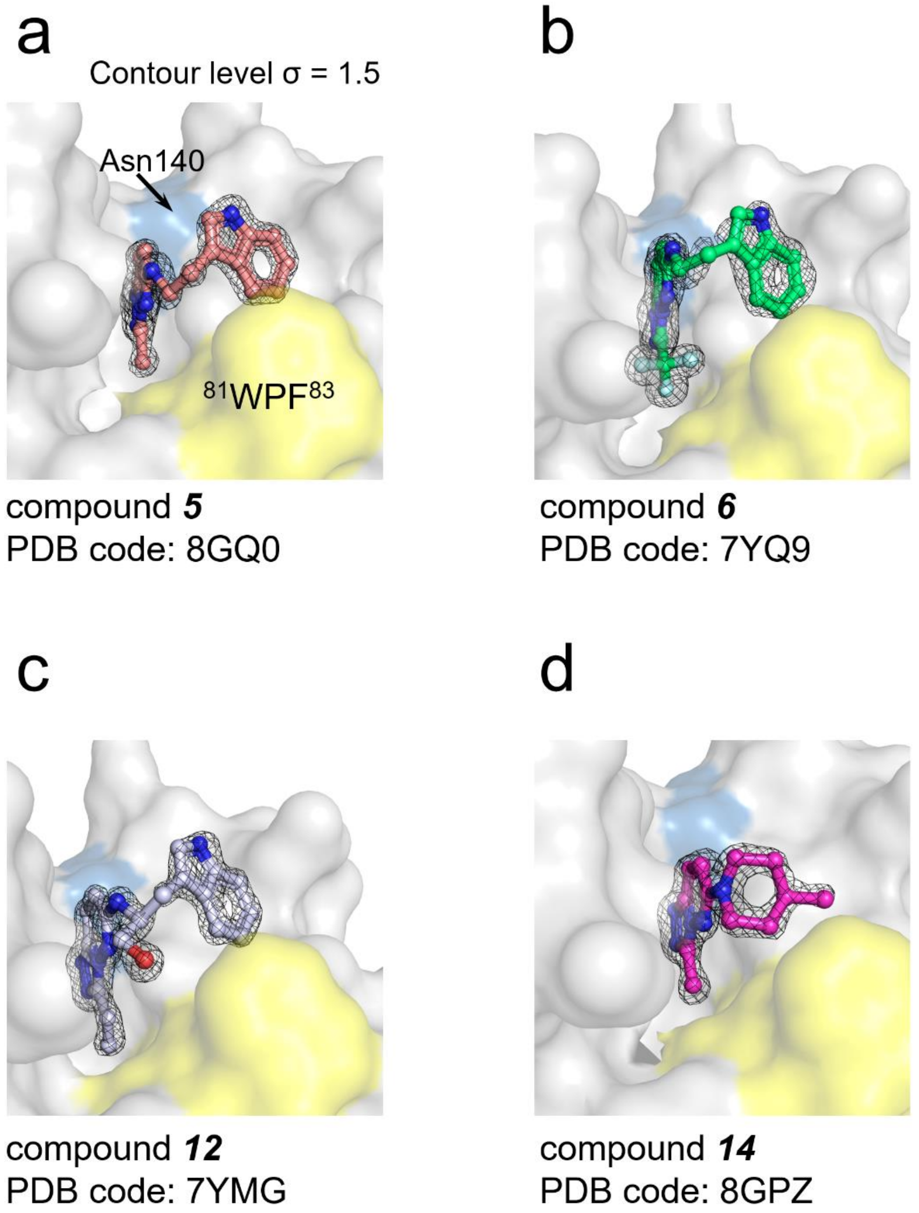

Supplementary Figure S4. Omit maps (2mFo-DFc) illustrate all compounds. (Contour level= 1.5σ). The figures were drawn using the program PyMOL version 2.0 (Schrödinger, New York, NY, USA).

Supplementary Table S1. Summary of conserved water molecules in the Kac binding site of BRD4 BD1 structures

**BD1 structures**		**PDB code**	**W1**	**W2**			**W3**	**W4**
				**W2-1**	**W2-2**	**W2-3**		
Apo		2OSS	O	O			O	O
mo-Kac	H3-K(ac)14	3JVK	O	O			O	O
mo-Kac	Rel Peptide	4KV1	O		O		O	O
mo-Kac	BAZ1B peptide/K221ac	5NNF	O	O	O		O	O
mo-Kac	SRPK1 peptide/K585ac	5NNG	O	O		O	O	O
mo-Kac	BCLTF1	7RJR	O	O			O	O
di-Kac	H4K8acK12ac	3UW9	O					O
di-Kac	H4K5acK8ac	3UVW	O	O			O	O
di-Kac	H4K12acK16ac	3UVX	O	O			O	O
di-Kac	H4K16acK20ac	3UVY	O			O	O	
di-Kac	H3K9ac/K14ac	5NNC	O	O				
di-Kac	H3K9ac/K14ac	5NND	O	O		O	O	O
di-Kac	TOP2A K1201ac/K1204ac	5NNE	O	O	O		O	O
di-Kac	ATRX peptide/K1030acK1033ac	6G0O	O		O		O	O
di-Kac	E2F1 peptide/K117acK120ac	6G0P	O		O		O	O
di-Kac	GATA1 peptide/K312acK315ac	6G0Q	O			O	O	O
di-Kac	POLR2A peptide/K775acK778ac	6G0R	O		O		O	O
di-Kac	SIRT7 peptide/K272acK275ac)	6G0S	O	O		O	O	O
di-Kac	cyclic peptide 3.1_3	6U6K	O		O		O	O
di-Kac	cyclic peptide 3.1_2_AcK7toA	6U8G			O		O	
di-Kac	cyclic peptide 3.2_1	6U8M	O		O			O
di-Kac	cyclic peptide 3.1_2_AcK5toA	6U72						
di-Kac	the cyclic peptide 3.1_2	6U74	O			O	O	
di-Kac	E2F1 peptide	6ULS	O	O			O	O
di-Kac	hnRNPK	7RJO	O	O	O	O	O	O
di-Kac	SHMT	7RJP	O	O		O	O	O
di-Kac	ILF3	7RLQ	O		O		O	O
tri-Kac	cyclic peptide 4.2_1	6ULV	O					

Supplementary Table S2. SAR study of [1,2,4]triazolo[4,3-*b*]pyridazine derivative compounds

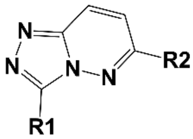

Compound No.	R1	R2	IC_{50} (μM) for BD1[a]
5	H_3C—	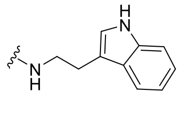	17.1±3.1
6	F_3C—	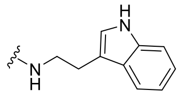	9.6±2.0
7	H_3C—	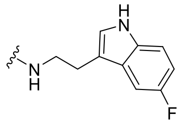	29.0±5.7
8	H_3C—	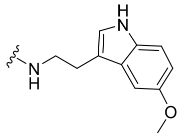	25.7±11.1
9	F_3C—	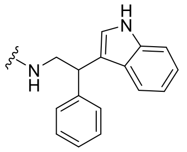	23.4±8.3
10	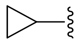	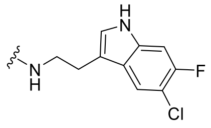	>32
11	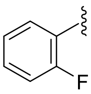	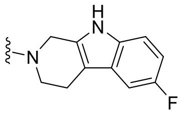	>32
12	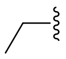	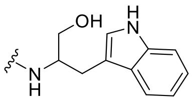	25.2±4.6
13	H_3C—	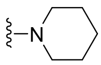	>32
14	H_3C—	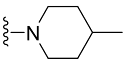	5.7±1.4

15	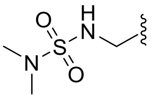	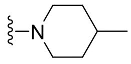	>32
16	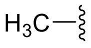	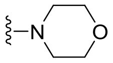	>32
17	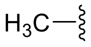	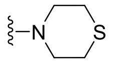	>32
18	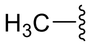	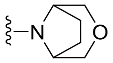	>32
19	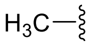	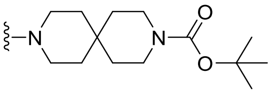	9.22±1.60
20	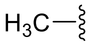	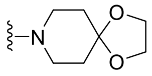	>32
21	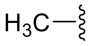	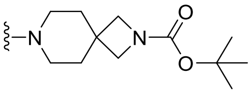	6.21±1.20
22	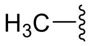	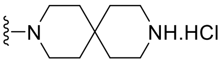	>32
23	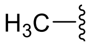	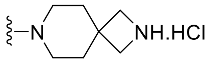	>32
24	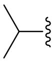	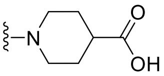	>32
25	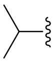	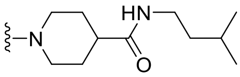	>32
26	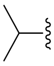	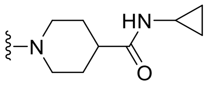	>32
27	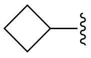	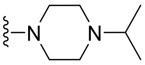	>32

28	H_3C—	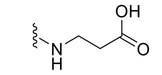	>32
29	H_3C—	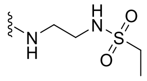	>32
30	H_3C—	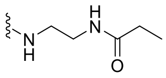	>32
31	H_3C—	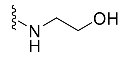	>32
32	H_3C—	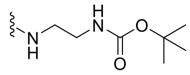	>32
33	H_3C—	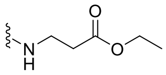	7.51±0.63
34	H_3C—	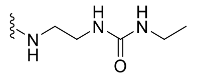	>32
35	H_3C—	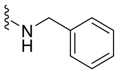	10.19±1.29
36	H_3C—	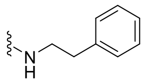	4.82±0.92
37	H_3C—	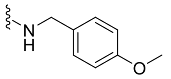	8.91±0.94
38	H_3C—	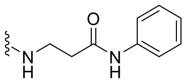	>32
39	H_3C—	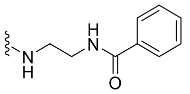	>32
40	H_3C—	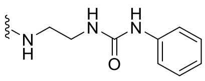	>32

[a]Inhibitory constant values (IC_{50}) are reported as the mean derived from three independent measurements.

Supplementary Table S3. Data collection and refinement statistics of crystal structural studies

Datasets	BD1-***5*** (PDB code: 8GQ0)	BD1-***6*** (PDB code: 7YQ9)	BD1-***12*** (PDB code: 7YMG)	BD1-***14*** (PDB code: 8GPZ)
**Diffraction-data statistics**				
X-ray source	PLS-11C	PLS-11C	PLS-7A	PLS-11C
Wavelength (Å)	0.979	0.979	0.979	0.979
Space group	$P2_12_12_1$	$P2_1$	$P2_1$	$P2_12_12_1$
Cell parameters				
a, b, c (Å)	32.28, 47.58, 79.34	47.50, 32.40, 79.47	47.57, 32.45, 79.63	32.160, 47.32, 79.54
α, β, γ (°)	90.00, 90.00, 90.00	90.00, 89.99, 90.00	90.00, 89.97, 90.00	90.00, 90.00, 90.00
Resolution range (Å)[a]	50–1.44 (1.46–1.44)	50–1.50 (1.53–1.50)	50–1.40 (1.42–1.40)	50–1.528 (1.56–1.53)
R_{sym} (%)[b]	6.6 (5.8)	6.1 (28.9)	5.2 (72.3)	9.5 (69.1)
$CC_{1/2}$	100 (83.8)	99.9 (96.2)	99.4 (70.8)	99.9 (78.2)
Mean I/σI	26 (2)	25.85 (5.4)	24.88 (1.9)	17.22 (2.0)
Redundancy	6.1 (6.1)	6.6 (5.8)	4.9 (4.5)	6.2 (5.1)
Completeness (%)	90.74 (59.90)	94.26 (78.39)	94.38 (70.62)	87.79 (56.58)
No. of unique reflections	20,708 (1,331)	37,047 (3,087)	45,701 (3,373)	16,654 (1,045)
**Refinement Statistics**				
Resolution range (Å)[a]	39.67–1.44 (1.49–1.44)	47.5–1.50 (1.55–1.50)	26.81–1.40 (1.45–1.40)	40.67–1.53 (1.585–1.53)
R_{work}/R_{free} (%)[c]	17.65/18.94	17.91/20.05	17.72/19.63	18.59/21.18
No. of nonhydrogen atoms	1,162	2,310	2,417	1,129
Protein	1,026	2,118	2,212	1,019
Solvent	88	134	138	86
Ligand	48	58	67	24
RMS deviation				
Bond length (Å)	0.007	0.007	0.006	0.007
Bond angles (°)	1.19	0.92	0.87	1.19
Ramachandran plot (%)				
Ramachandran favored (%)	98.36	98.35	98.35	98.36
Ramachandran allowed (%)	1.64	1.65	1.65	1.64
Ramachandran outliers (%)	0	0	0	0
Rotamer outliers (%)	0	0	0.79	0
Clash score	1.90	2.55	2.42	2.93

[a]Value in parentheses are for the highest-resolution shell.

[b]$R_{sym} = \Sigma_h \Sigma_i | I(h)_i - < I(h) > | / \Sigma_h \Sigma_i I(h)_i$, where I(h) is the intensity for reflection h, Σ_h is the sum for all reflections, and Σ_i is the sum for i measurements of reflection h.

[c]$R = \Sigma | |F_{obs}| - |F_{calc}| | / \Sigma |F_{obs}|$, where R_{free} is calculated for a randomly chosen 5% of reflections, which were not used for structure refinement, and R_{work} is calculated for the remaining reflections.

Chemical experiment

6-chloro-3-methyl-[1,2,4]triazolo[4,3-*b*]pyridazine (*3*)

Compound ***1*** (3,6-dichloropyridazine; 500 mg, 3.35 mmol) and acetohydrazide (248 mg, 3.35 mmol) were dissolved in anhydrous n-BuOH (5 mL). The resulting solution was stirred at 120 °C for 16 h, and the reaction progress was monitored by TLC (eluent condition: 50% EtOAc in hexanes). The reaction was quenched by adding H_2O (30 mL) and partitioned with EtOAc (100 mL). The organic layer was washed with H_2O (2 × 30 mL) and brine (20 mL). The organic layer was collected, dried over anhydrous Na_2SO_4, filtered, concentrated, and purified by silica column chromatography (3% MeOH in DCM). This purification process yielded compound ***3*** with a yield of 42%, corresponding to 240 mg as a brown solid. ^{1}H NMR (500 MHz, DMSO-d_6) δ 8.42 (d, J = 9.6 Hz, 1H), 7.47 (d, J = 9.6 Hz, 1H), 2.69 (s, 3H). ESI-MS, m/z = 169.1 $[M+H]^+$.

N-(2-(1H-indol-3-yl)ethyl)-3-methyl-[1,2,4]triazolo[4,3-*b*]pyridazin-6-amine (*5*)

Compound ***3*** (50 mg, 0.29 mmol) and 2-(1H-indol-3-yl)ethan-1-amine (46 mg, 0.29 mmol) were dissolved in anhydrous EtOH (2 mL). KI (5 mg, 0.03 mmol), conc. HCl (concentrated HCl, 12 N; 1 µL, 0.01 mmol) and Et_3N (83 µL, 0.60 mmol) were added to the reaction flask. The reaction mixture was stirred at 80 °C for 24 h, and the reaction progress was monitored by TLC (eluent condition: 4% MeOH in DCM). The reaction was quenched by adding H_2O (10 mL) and partitioned with DCM (30 mL). The organic layer was washed with H_2O (20 mL) and brine (20 mL). The organic layer was collected, dried over anhydrous Na_2SO_4, filtered, concentrated, and purified by silica column chromatography (4% MeOH in DCM). This purification process yielded compound ***5*** with a yield of 48%, corresponding to 42 mg as a light-brown solid. ^{1}H NMR (400 MHz, DMSO-d_6) δ 10.84 (s, 1H), 7.84 (d, J = 9.7 Hz, 1H), 7.64 (d, J = 7.8 Hz, 1H), 7.46 (t, J = 5.5 Hz, 1H), 7.36 (d, J = 8.1 Hz, 1H), 7.21 (d, J = 2.4 Hz, 1H), 7.08 (t, J = 7.5 Hz, 1H), 7.00 (t, J = 7.4 Hz, 1H), 6.77 (d, J = 9.8 Hz, 1H), 3.55 (q, J = 6.8 Hz, 2H), 3.05 (q, J = 8.6, 7.4 Hz, 2H), 2.55 (s, 3H); ^{13}C NMR (100 MHz, DMSO-d_6) δ 154.31, 145.65, 142.74, 136.75, 127.72, 123.92, 123.37, 121.41, 118.78, 118.70, 116.97, 112.26, 111.89, 70.25, 46.26, 42.29, 24.54, 9.76, 9.12; ESI-MS, m/z = 293.5 $[M+H]^+$.

3-Methyl-6-(piperidin-1-yl)-[1,2,4]triazolo[4,3-*b*]pyridazine (*13*)

Compound ***3*** (150 mg, 0.9 mmol) and piperidine (151 mg, 1.78 mmol) were dissolved in anhydrous EtOH (3 mL). KI (15 mg, 0.09 mmol) and conc. HCl (4 µL, 0.04 mmol) were added to the reaction flask. The reaction mixture was stirred at 80 °C for 24 h, and the reaction progress was monitored by TLC (eluent condition: 5% MeOH in DCM). The reaction solvent was removed under vacuum, and the crude product was purified by silica column chromatography (1% MeOH in DCM). This purification process yielded compound ***13*** with a yield of 52% corresponding to 100 mg with a light brown solid.

^1H NMR (400 MHz, DMSO-d_6) δ 7.95 (d, *J* = 10.2 Hz, 1H), 7.30 (d, *J* = 10.2 Hz, 1H), 3.54 (dd, *J* = 6.2, 3.8 Hz, 4H), 1.64–1.54 (m, 6H); ^{13}C NMR (100 MHz, DMSO-d_6) δ 155.30, 145.68, 142.26, 124.46, 114.51, 70.26, 46.79, 29.48, 25.35, 24.35, 9.67; ESI-MS, m/z = 218.3 $[M+H]^+$.

3-Methyl-6-(4-methylpiperidin-1-yl)-[1,2,4]triazolo[4,3-*b*]pyridazine (*14*)

Compound ***3*** (200 mg, 1.1 mmol) and 4-methylpiperidine (235 mg, 2.3 mmol) were dissolved in anhydrous EtOH (3 mL). KI (19.6 mg, 0.12 mmol) and conc. HCl (5 µL, 0.06 mmol) were added to the reaction flask. The reaction mixture was stirred at 80 °C for 24 h, and the reaction progress was monitored by TLC (eluent condition: 50% MeOH in DCM). The reaction was quenched by adding H_2O (15 mL) and partitioned with DCM (30 mL). The organic layer was washed with H_2O (20 mL) and brine (20 mL). The organic layer was collected, dried over anhydrous Na_2SO_4, filtered, concentrated, and purified by silica column chromatography (4% MeOH in DCM). This purification process yielded compound ***14*** with a yield of 22%, corresponding to 60 mg as a light-brown solid. ^1H NMR (400 MHz, DMSO-d_6) δ 7.96 (d, *J* = 10.1 Hz, 1H), 7.31 (d, *J* =10.2 Hz, 1H), 4.19 (d, *J* = 13.1 Hz, 2H), 2.90 (td, *J* = 12.8, 2.6 Hz, 2H),2.53 (s, 3H), 1.77–1.54 (m, 3H), 1.15(qd, *J* = 12.3, 3.9 Hz, 2H), 0.91 (d, *J* =6.5 Hz, 3H); ^{13}C NMR (100 MHz, DMSO-d_6) δ 155.26, 145.67, 142.27, 124.49, 114.55, 46.16, 33.55, 30.71, 22.09, 9.69; ESI-MS, m/z = 232.4 $[M+H]^+$.

Tert-butyl ((6-chloro-[1,2,4]triazolo[4,3-*b*]pyridazin-3-yl)methyl)carbamate (*4a*)

Compound ***3*** (500 mg, 3.35 mmol) and tert-butyl (2-hydrazineyl-2-oxoethyl)carbamate (635 mg, 3.35 mmol) were dissolved in anhydrous EtOH (5 mL). *p*-TSA (32 mg, 0.17 mmol) was added to the reaction flask, and then the reaction was stirred at 80 °C for 14 h. The reaction progress was monitored by TLC (eluent Condition 5% MeOH in DCM). The reaction was quenched by adding water (20 mL) and partitioned with EtOAc (30 ml). The organic layer was collected, dried over anhydrous Na_2SO_4, filtered, concentrated, and purified by silica column chromatography (2% MeOH in DCM). This purification process yielded intermediate ***4a*** with a yield of 23%, corresponding to 220 mg as a dark brown solid. ^1H NMR (300 MHz, DMSO-d_6) δ 8.46 (d, *J* = 9.7 Hz, 1H), 7.51 (d, *J*= 9.7 Hz, 2H), 4.64 (d, *J* = 5.6 Hz, 2H), 1.39 (d, *J* = 4.6 Hz, 11H).

Tert-butyl ((6-(4-methylpiperidin-1-yl)-[1,2,4]triazolo[4,3-*b*]pyridazin-3-yl)methyl)carbamate (*4b*)

Compound ***4*a** (200 mg, 0.70 mmol) and 4-methylpiperidine (140 mg, 1.41 mmol) were dissolved in anhydrous EtOH (2 mL) in a reaction flask, KI (12 mg, 0.07 mmol) and conc. HCl (3 µL, 0.03 mmol) were added, and then the reaction was stirred at 80 °C for 24 h. The reaction progress was monitored by TLC (eluent Condition 5% MeOH in DCM). The reaction solvent was removed under vacuum, and the crude product was purified by silica column chromatography (4% MeOH in DCM). This purification

process yielded compound ***4b*** with a yield of 23% (58 mg). ^{1}H NMR (400 MHz, DMSO-d_6) δ 7.99 (d, *J* = 10.2 Hz, 1H), 7.39–7.28 (m, 2H), 4.55 (d, *J* = 5.7 Hz, 2H), 4.23 (dt, *J* = 13.4, 3.1 Hz, 2H), 2.90 (td, *J* = 12.8, 2.5 Hz, 2H), 1.69 (d, *J* = 12.1 Hz, 2H), 1.62 (td, *J* = 6.7,3.2 Hz, 1H), 1.39 (s, 8H), 1.17 (dtt, *J* = 23.7, 11.4, 5.5 Hz, 3H), 0.92 (d, *J* = 6.2 Hz, 3H); ^{13}C NMR (100 MHz, DMSO-d_6) δ 155.87, 155.12, 146.53, 142.64, 124.50, 114.95, 78.47, 46.10, 34.61, 33.61, 30.72, 28.67, 22.11; ESI-MS, m/z = 347.7 $[M+H]^+$.

(6-(4-Methylpiperidin-1-yl)-[1,2,4]triazolo[4,3-*b*]pyridazin-3-yl)methanamine (*4c*)

Compound ***4*b** (160 mg, 0.46 mmol) was dissolved in DCM (1 mL) and cooled to approximately 5 °C. TFA solution (2 mL with 1 mL of DCM) was added dropwise into the reaction flask. After the addition reaction was stirred for 1 h at RT, the reaction progress was monitored by TLC (eluent condition 5% MeOH in DCM). The reaction solvent was distilled out, and the product was dried under vacuum. This purification process yielded compound ***4c*** in quantitative yield (400 mg).

***N*-((6-(4-methylpiperidin-1-yl)-[1,2,4]triazolo[4,3-*b*]pyridazin-3-yl)methyl)*N*,*N*-dimethyl-2-sulfonamide (*15*)**

Compound ***4*c** (100 mg, 0.40 mmol) and anhydrous pyridine (2 mL) were placed in a reaction flask, stirred and cooled to 0 °C. *N*,*N*-Dimethylsulfamoyl chloride (116 mg, 0.81 mmol) was added dropwise into the reaction flask and then stirred at RT for 2 h, and the reaction progress was monitored by TLC (eluent condition 5% MeOH in DCM). The reaction was quenched by adding water (20 mL) and partitioned with EtOAc (25 mL). The separated organic layer was washed with 1 N HCl solution and then brine solution, and the organic layer was collected, dried over anhydrous Na_2SO_4, filtered, concentrated, and purified by silica column chromatography (4% MeOH in DCM). This purification process yielded compound ***15*** with a yield of 36% (52 mg). ^{1}H NMR (400 MHz, DMSO-d_6) δ 8.02 (d, *J* = 9.9 Hz, 1H), 7.80 (s, 1H), 7.39 (d, *J* = 10.2 Hz, 1H), 4.60–4.37 (m, 2H), 4.27 (d, *J* = 13.3 Hz, 2H), 2.92 (t, *J* = 12.7 Hz, 2H), 2.61 (s, 6H), 1.68 (t, *J* = 16.2 Hz, 3H), 1.15 (q, *J* = 12.9 Hz, 2H), 0.92 (d, *J* = 6.2 Hz, 3H); ^{13}C NMR (100 MHz, DMSO-d_6) δ 155.16, 145.71, 142.60, 124.51, 115.19, 46.14, 37.92, 36.57, 33.60, 30.76, 22.14. ESI-MS = 354.5 $[M+H]^+$.

4-(3-Methyl-[1,2,4]triazolo[4,3-*b*]pyridazin-6-yl)morpholine (*16*)

Compound ***3*** (150 mg, 0.89 mmol) and morpholine (155 mg, 1.78 mmol) were dissolved in anhydrous EtOH (3 mL) in a reaction flask. KI (15 mg, 0.09 mmol) and conc. HCl (4 µL, 0.04 mmol) were charged in the reaction flask, and the reaction was stirred at 80 °C for 24 h. The reaction progress was monitored by TLC (eluent condition 5% MeOH in DCM). The reaction solvent was removed under vacuum, and the crude product was purified by silica column chromatography (1% MeOH in DCM). This purification process yielded compound ***16*** with a yield of 61% (120 mg). ^{1}H NMR (400 MHz, DMSO-d_6) δ 8.03 (d,

J = 10.1 Hz, 1H), 7.30 (d, J = 10.1 Hz, 1H), 3.77–3.69 (m, 4H), 3.51 (dd, J = 5.8, 4.1 Hz, 4H), 2.55 (s, 3H); ^{13}C NMR (101 MHz, DMSO-d_6) δ 155.57, 145.82, 142.47, 124.77, 114.09, 66.09, 46.06, 9.67; ESI-MS, m/z = 220.4 [M+H]$^+$.

4-(3-Methyl-[1,2,4]triazolo[4,3-*b*]pyridazin-6-yl)thiomorpholine (*17*)

Compound ***3*** (150 mg, 0.89 mmol), thiomorpholine (183 mg, 1.78 mmol) and anhydrous EtOH (3 mL) were charged in a reaction flask and stirred. KI (15 mg, 0.09 mmol) and conc. HCl (4 µL, 0.04 mmol) were added into the reaction flask. The reaction mixture was stirred at 83 °C in a sealed tube for 24 h, and the reaction progress was monitored by TLC (eluent condition 5% MeOH in DCM). The reaction solvent was removed under vacuum, and the crude product was purified by silica column chromatography (1% MeOH in DCM). This purification process yielded compound ***17*** with a yield of 38%, corresponding to 80 mg of an off-white solid. ^{1}H NMR (400 MHz, DMSO-d_6) δ 8.00 (d, J = 10.2 Hz, 1H), 7.31 (d, J = 10.2 Hz, 1H), 3.95–3.85 (m, 4H), 2.72–2.64 (m, 4H), 2.54 (s, 3H). ^{13}C NMR (101 MHz, DMSO-d_6) δ 154.62, 145.77, 142.25, 124.83, 114.42, 48.61, 25.79, 9.67; ESI-MS, m/z = 236.3 [M+H]$^+$.

8-(3-Methyl-[1,2,4]triazolo[4,3-*b*]pyridazin-6-yl)-3-oxa-8-azabicyclo[3.2.1] octane (*18*)

Compound ***3*** (100 mg, 0.60 mmol), 8-(3-methyl-[1,2,4]triazolo[4,3-*b*]pyridazin-6-yl)-3-oxa-8-azabicyclo[3.2.1]octane (97 mg, 0.65 mmol) and anhydrous EtOH (3 mL) were added in a reaction flask and stirred. KI (10 mg, 0.06 mmol) and conc. HCl (3 µL, 0.03 mmol) were added into the reaction flask. The reaction mixture was stirred at 80 °C for 24 h, and the reaction progress was monitored by TLC (eluent condition 5% MeOH in DCM). The reaction solvent was removed under vacuum, and the crude product was purified by silica column chromatography (1% MeOH in DCM). This purification process yielded compound ***18*** with a yield of 41% (60 mg). ^{1}H NMR (400 MHz, DMSO-d_6) δ 8.03 (d, J = 10.0 Hz, 1H), 7.22 (d, J = 10.0 Hz, 1H), 4.50 (s, 2H), 3.71 (d, J = 10.9 Hz, 2H), 3.55 (d, J = 11.0 Hz, 2H), 2.54 (s, 3H), 1.97 (dt, J = 12.2, 8.5 Hz, 4H); ^{13}C NMR (101 MHz, DMSO-d_6) δ 153.22, 145.75, 142.50, 125.03, 115.37, 70.27, 56.28, 46.26, 26.82, 9.65, 9.11; ESI-MS, m/z = 246.4 [M+H]$^+$.

Tert-butyl 9-(3-methyl-[1,2,4]triazolo[4,3-*b*]pyridazin-6-yl)-3,9-diazaspiro[5.5]undecane-3-carboxylate (*19*)

Compound ***3*** (250 mg, 1.48 mmol) and tert-butyl 3,9-diazaspiro[5.5]undecane-3-carboxylate (754 mg, 2.96 mmol) were dissolved in anhydrous EtOH (3 mL). KI (24 mg, 0.14 mmol) and conc. HCl (7 µL, 0.07 mmol) was added to the reaction mixture. The reaction was stirred at 80 °C for 72 h, and the reaction progress was monitored by TLC (eluent condition: 10% MeOH in DCM). The reaction solvent was removed under vacuum, and the crude product was purified by silica column chromatography (3% MeOH in DCM). This purification process yielded compound ***19*** with a yield of 17% (97 mg). ^{1}H NMR

(400 MHz, DMSO-d_6) δ 7.96 (d, J = 10.1 Hz, 1H), 7.32 (d, J = 10.2 Hz, 1H), 3.58–3.49 (m, 4H), 3.35–3.27 (m, 4H), 2.54 (s, 3H), 1.58–1.50 (m, 4H), 1.39 (s, 13H); ^{13}C NMR (101 MHz, DMSO-d_6) δ 155.38, 154.43, 145.69, 142.30, 124.47, 114.50, 78.90, 41.60, 35.11, 34.53, 30.17, 28.58, 9.69; ESI-MS, m/z = 387.7[M+H]$^+$.

8-(3-Methyl-[1,2,4]triazolo[4,3-*b*]pyridazin-6-yl)-1,4-dioxa-8-azaspiro[4.5]decane (*20*)

Compound ***3*** (150 mg, 0.89 mmol) and 1,4-dioxa-8-azaspiro[4.5]decane (183 mg, 1.78 mmol) were dissolved in anhydrous EtOH (3 mL). KI (15 mg, 0.09 mmol) and conc. HCl (4 µL, 0.04 mmol) was added to the reaction flask. The reaction mixture was stirred at 80 °C for 24 h, and the reaction progress was monitored by TLC (eluent condition: 5% MeOH in DCM). The reaction solvent was removed under vacuum, and the crude product was purified by silica column chromatography (1% MeOH in DCM). This purification process yielded compound ***20*** with a yield of 24% (58 mg). ^{1}H NMR (400 MHz, DMSO-d_6) δ 7.99 (d, J = 10.1 Hz, 1H), 7.35 (d, J = 10.1 Hz, 1H), 3.93 (s, 4H), 3.68–3.61 (m, 4H), 2.54 (s, 3H), 1.75–1.68 (m, 4H); ^{13}C NMR (101 MHz, DMSO-d_6) δ 154.90, 145.75, 142.28, 124.70, 114.51, 106.75, 64.30, 44.27, 34.35; ESI-MS, m/z = 276.$^{5\ [M+H]+}$.

Tert-butyl 7-(3-methyl-[1,2,4]triazolo[4,3-*b*]pyridazin-6-yl)-2,7-diazaspiro[3.5]nonane-2-carboxylate (*21*)

Compound ***3*** (250 mg, 1.48 mmol) and tert-butyl 2,7-diazaspiro[3.5]nonane-2-carboxylate (671 mg, 2.96 mmol) were dissolved in anhydrous EtOH (3 mL). KI (24 mg, 0.14 mmol) and conc. HCl (7 µL, 0.07 mmol) was added to the reaction flask. The reaction mixture was stirred at 80 °C for 72 h, and the reaction progress was monitored by TLC (eluent condition: 10% MeOH in DCM). The reaction solvent was removed under vacuum, and the crude product was purified by a silica column (3% MeOH in DCM). This purification process yielded compound ***21*** with a yield of 17% (93 mg). ^{1}H NMR (400 MHz, DMSO-d_6) δ 7.97 (d, J = 10.1 Hz, 1H), 7.34 (d, J = 10.2 Hz, 1H), 3.69–3.46 (m, 8H), 2.54 (s, 3H), 1.76 (t, J = 5.5 Hz, 4H), 1.38 (s, 9H); ^{13}C NMR (101 MHz, DMSO-d_6) δ 156.13, 155.23, 145.71, 142.28, 124.61, 114.55, 78.91, 43.18, 34.46, 33.69, 28.56, 9.69; ESI-MS. m/z = 359.6 [M+H]$^+$.

3-(3-Methyl-[1,2,4]triazolo[4,3-*b*]pyridazin-6-yl)-3,9-diazaspiro[5.5]undecane hydrochloride (*22*)

Compound ***19*** (200 mg, 0.51 mmol) was dissolved in 1,4-dioxane (4 mL) and cooled to 0 °C. HCl (4 M) in dioxane (3 mL, 0.01 mmol) was added to the reaction flask and stirred for 3 h at RT, and the reaction progress was monitored by TLC (eluent condition: 10% MeOH in DCM). The reaction solvent was removed under vacuum, and the product was obtained. After that, the product was triturated with diethyl ether. This purification process yielded compound ***22*** with a yield of 74% (110 mg). ^{1}H NMR (400 MHz, DMSO-d_6) δ 9.19 (s, 2H), 8.22 (d, J = 10.3 Hz, 1H), 7.83 (d, J = 10.3 Hz, 1H), 3.66 (ddd, J = 7.9, 4.6, 2.6 Hz, 4H), 3.52–3.44 (m, 1H), 3.03 (dq, J = 7.6, 3.9 Hz, 4H), 2.68 (s, 3H), 1.71 (t, J = 5.7

Hz, 4H), 1.63–1.53 (m, 4H); ^{13}C NMR (101 MHz, DMSO-d_6) δ 156.00, 145.88, 140.31, 122.64, 119.84, 72.61, 70.98, 60.62, 44.11, 41.42, 34.35, 31.65, 29.45, 9.31; ESI-MS, m/z = 287.5 $[M+H]^+$.

3-Methyl-6-(2,7-diazaspiro[3.5]nonan-7-yl)-[1,2,4]triazolo[4,3-*b*]pyridazine hydrochloride (*23*)

Compound ***21*** (200 mg, 0.51 mmol) was dissolved in 1,4-dioxane (4 mL) and cooled to 0 °C. HCl (4 M) in dioxane (3 mL, 0.01 mmol) was added to the reaction flask and stirred for 3 h at RT, and the reaction progress was monitored by TLC (eluent condition: 10% MeOH in DCM). The reaction solvent was removed under vacuum to afford the crude product, and the product was triturated with diethyl ether. This purification process yielded compound ***23*** with a yield of 70% (101 mg). ^{1}H NMR (400 MHz, DMSO-d_6) δ 9.65 (s, 2H), 8.23 (d, *J* = 10.1 Hz, 1H), 7.83 (d, *J* = 10.2 Hz, 1H), 3.76 (t, *J* = 6.1 Hz, 4H), 3.64 (dd, *J* = 7.3, 4.0 Hz, 4H), 2.68 (s, 3H), 1.91 (dd, *J* = 7.2, 3.9 Hz, 4H); ^{13}C NMR (101 MHz, DMSO-d_6) δ 155.94, 145.94, 140.35, 122.84, 119.75, 72.60, 70.98, 60.62, 54.38, 44.11, 42.60, 36.71, 33.74, 9.28; ESI-MS, m/z = 259.5 $[M+H]^+$.

3-((3-Methyl-[1,2,4]triazolo[4,3-*b*]pyridazin-6-yl)amino)propanoic acid (*28*)

Compound ***33*** (1.2 g, 4.81 mmol) was dissolved in a mixture of solvents THF (18 mL) and water (6 mL); LiOH. H_2O (606 mg, 14.44 mmol) was added to the reaction flask, and the reaction was stirred for 2 h at RT. The reaction progress was monitored by TLC (eluent condition: 10% MeOH in DCM). The reaction mixture was diluted with water (3 mL), and the pH was adjusted to 4 ~ 5 with 1 N HCl; multiple extractions with ethyl acetate were not successful, as more product remained in the aqueous layer. Therefore, reaction solvents were removed under vacuum, and the crude product was purified with silica column chromatography (8% MeOH in DCM). This purification process yielded compound ***28*** with a yield of 67% (710 mg). ^{1}H NMR (500 MHz, DMSO-d_6) δ 7.84 (d, *J* = 9.8 Hz, 1H), 7.64 (t, *J* = 5.4 Hz, 1H), 6.83 (d, *J* = 9.8 Hz, 1H), 3.45 (q, *J* = 6.6 Hz, 2H), 2.60 (t, *J* = 6.8 Hz, 2H), 2.52 (s, 3H); ^{13}C NMR (100 MHz, DMSO-d_6) δ 173.57, 154.24, 145.66, 142.69, 123.88, 116.91, 70.24, 49.01, 37.41, 33.45, 9.70; ESI-MS, m/z = 222.13 $[M+H]^+$.

N-(2-((3-methyl-[1,2,4]triazolo[4,3-*b*]pyridazin-6-yl)amino)ethyl)ethanesulfonamide (*29*)

Intermediate ***32*a** (180 mg, 0.79 mmol), DIPEA (356 mg, 2.76 mmol, 0.5 mL) and anhydrous EtOH (2 mL) were added to the reaction flask, stirred, and cooled to 0 °C. Ethanesulfonyl chloride (152 mg, 1.18 mmol, 0.1 mL) was added dropwise to the reaction mixture and then stirred for 2 h at RT, and the reaction progress was monitored by TLC (eluent condition: 15% (10% ammonia in MeOH) in DCM). The reaction solvent was removed under vacuum to obtain the crude product, and further purification was performed by prep-TLC (10% in DCM (10% ammonia in MeOH)). This purification process yielded compound ***29*** with a yield of 18% (40 mg). ^{1}H NMR (400 MHz, DMSO-d_6) δ 7.86 (dd, *J* = 9.8, 1.2 Hz, 1H), 7.48 (t, *J* = 5.6 Hz, 1H), 7.21 (t, *J* = 5.8 Hz, 1H), 6.77 (dd, *J* = 9.8, 1.2 Hz, 1H), 3.38 (q, *J*

= 6.4 Hz, 2H), 3.21 (t, J = 6.3 Hz, 2H), 3.07–2.98 (m, 2H), 2.54–2.51 (m, 3H), 1.23–1.16 (m, 3H); ^{13}C NMR (100 MHz, DMSO-d_6) δ 154.22, 145.64, 142.70, 124.08, 116.84, 45.92, 41.71, 41.01, 9.65, 8.57; ESI-MS, m/z = 285.4 $[M+H]^+$.

N-(2-((3-methyl-[1,2,4]triazolo[4,3-*b*]pyridazin-6-yl)amino)ethyl)propionamide (*34*)

Propionic acid (32 mg, 0.43 mmol), TEA (221 mg, 2.19 mmol, 0.3 mL) and HATU (199 mg, 0.52 mmol) were dissolved in anhydrous DMF (2 mL) sequentially and stirred for 5 min at RT. Intermediate ***32*a** (100 mg, 0.43 mmol) was added to the reaction mixture, and the reaction was stirred for 2 h at RT. Reaction progress monitored by TLC (eluent condition: 15% (10% Ammonia in MeOH) in DCM). The reaction was quenched by adding H_2O (5 ml) and partitioned with 5% MeOH in DCM. The organic layer was washed with brine (10 mL). The organic layer was collected, dried over anhydrous Na_2SO_4, filtered, concentrated and purified by silica column chromatography (3% (10% ammonia in MeOH) in DCM). This purification process yielded compound ***34*** with a yield of 29%, corresponding to 32 mg as an off-white solid. ^{1}H NMR (400 MHz, DMSO-d_6) δ 7.92–7.81 (m, 2H), 7.36 (d, J = 5.3 Hz, 1H), 6.73 (d, J = 9.8 Hz, 1H), 3.33–3.26 (m, 4H), 2.52 (s, 3H), 2.08 (q, J = 7.6 Hz, 2H), 0.99 (t, J = 7.6 Hz, 3H). ^{13}C NMR (100 MHz, DMSO-d_6) δ 173.61, 154.33, 145.61, 142.70, 123.99, 116.85, 40.98, 37.72, 28.95, 10.31, 9.68; ESI-MS, m/z = 249.4 $[M+H]^+$.

2-((3-Methyl-[1,2,4]triazolo[4,3-*b*]pyridazin-6-yl)amino)ethan-1-ol (*31*)

Compound ***3*** (100 mg, 0.60 mmol) and ethanolamine (108 mg, 1.78 mmol) were dissolved in anhydrous EtOH (3 mL). KI (10 mg, 0.06 mmol) and conc. HCl (3 µL, 0.03 mmol) were added to the reaction flask. The reaction mixture was stirred at 80 °C for 72 h, and the reaction progress was monitored by TLC (eluent condition: 10% MeOH in DCM). The reaction solvent was removed under vacuum, and the crude product was purified by silica column chromatography (3% MeOH in DCM). This purification process yielded compound ***31*** in 57% yield (65 mg). ^{1}H NMR (400 MHz, DMSO-d_6) δ 7.83 (d, J = 9.8 Hz, 1H), 7.34 (t, J = 5.4 Hz, 1H), 6.81 (d, J = 9.9 Hz, 1H), 4.81 (s, 1H), 3.63 (t, J = 5.9 Hz, 2H), 3.34 (q, J = 5.7 Hz, 2H); ^{13}C NMR (100 MHz, DMSO-d_6) δ 154.47, 145.59, 142.70, 123.81, 117.00, 59.28, 44.00, 9.69; ESI-MS, m/z = 194.3 $[M+H]^+$.

Tert-butyl(2-((3-methyl-[1,2,4]triazolo[4,3-*b*]pyridazin-6-yl)amino)ethyl)carbamate (*32*)

Compound ***3*** (1 g, 5.93 mmol) and tert-butyl (2-aminoethyl)carbamate (1.4 g, 1.42 mmol) were dissolved in anhydrous EtOH (10 mL). KI (98 mg, 0.59 mmol) and conc. HCl (30 µL, 0.3 mmol) were added to the reaction flask. The reaction was stirred at 80 °C for 72 h, and the reaction progress was monitored by TLC (eluent condition: 5% MeOH in DCM). The reaction solvent was removed under vacuum, and the crude product was purified by silica column chromatography (2% MeOH in DCM). This purification process yielded compound ***32*** with a yield of 81% (1.4 g). ^{1}H NMR (400 MHz, DMSO-

d_6) δ 7.84 (d, *J* = 9.8 Hz, 1H), 7.34 (t, *J* = 5.5 Hz, 1H), 6.89 (t, *J* = 5.8 Hz, 1H), 6.73 (d, *J* = 9.8 Hz, 1H), 3.30 (q, *J* = 6.3, 5.8 Hz, 2H), 3.20 (q, *J* = 6.8, 6.4 Hz, 2H), 2.52 (s, 3H), 1.38 (d, *J* = 11.5 Hz, 9H); ^{13}C NMR (100 MHz, DMSO-d_6) δ 156.16, 154.31, 145.61, 142.69, 123.94, 116.88, 78.14, 41.29, 39.08, 38.25, 28.67, 28.64, 9.65; ESI-MS, m/z = 293.5 $[M+H]^+$.

Ethyl 3-((3-methyl-[1,2,4]triazolo[4,3-*b*]pyridazin-6-yl)amino)propanoate (*33*)

Compound ***3*** (1.5 g, 8.90 mmol) and ethyl 3-aminopropanoate (1.3 g, 8.90 mmol) were dissolved in anhydrous EtOH (10 mL). KI (148 mg, 0.9 mmol) and conc. HCl (40 µL, 0.4 mmol) were added to the reaction flask. The reaction was stirred at 80 °C for 72 h, and the reaction progress was monitored by TLC (eluent condition: 10% MeOH in DCM). The reaction solvent was removed under vacuum, and the crude product was purified by silica column chromatography (3% MeOH in DCM). This purification process yielded compound ***33*** with a yield of 54% (1.4 g). ^{1}H NMR (400 MHz, DMSO-d_6) δ 7.85 (d, *J* = 9.8 Hz, 1H), 7.44 (t, *J* = 5.5 Hz, 1H), 6.74 (d, *J* = 9.9 Hz, 1H), 4.08 (q, *J* = 7.1 Hz, 2H), 3.50 (q, *J* = 6.3 Hz, 2H), 2.67 (t, *J* = 6.6 Hz, 2H), 2.52 (s, 3H), 1.17 (t, *J* = 7.1 Hz, 3H); ^{13}C NMR (100 MHz, DMSO-d_6) δ 171.95, 154.12, 145.65, 142.66, 124.08, 116.71, 60.45, 46.26, 37.22, 33.18, 14.52, 9.66, 9.11; ESI-MS, m/z= 250.4 $[M+H]^+$.

1-Ethyl-3-(2-((3-methyl-[1,2,4]triazolo[4,3-*b*]pyridazin-6-yl)amino)ethyl)urea (*34*)

Compound ***32*a** (100 mg, 0.43 mmol) and DIPEA (113 mg, 0.87 mmol) was mixed with acetonitrile (1 mL) and stirred for 5 min at RT. Ethylisocyanate (34 mg, 0.48 mmol, 38 µL) was added dropwise into the reaction mixture and stirred at RT for 5 h, and the reaction progress was monitored by TLC (eluent condition: 15% (10% ammonia in MeOH) in DCM). The reaction mixture was filtered, and the product was washed with cold acetonitrile. This purification process yielded compound ***34*** with a yield of 50%, corresponding to 58 mg as a white solid. ^{1}H NMR (400 MHz, DMSO-d_6) δ 7.84 (d, *J* = 9.8 Hz, 1H), 7.37 (t, *J* = 4.8 Hz, 1H), 6.74 (d, *J* = 9.8 Hz, 1H), 5.92 (dt, *J* = 24.1, 5.7 Hz, 2H), 3.27 (dt, *J* = 8.5, 5.3 Hz, 4H), 3.01 (p, *J* = 6.8 Hz, 2H), 0.97 (t, *J* = 7.2 Hz, 3H); ^{13}C NMR (100 MHz, DMSO-d_6) δ 158.59, 154.39, 145.63, 142.69, 123.96, 116.82, 42.07, 38.57, 34.56, 16.11, 9.68; ESI-MS= m/z, 264.4 $[M+H]^+$.

N-benzyl-3-methyl-[1,2,4]triazolo[4,3-*b*]pyridazin-6-amine (*35*)

Compound ***3*** (100 mg, 0.6 mmol) and benzylamine (127 mg, 1.18 mmol) were dissolved in anhydrous EtOH (2 mL). KI (10 mg, 0.06 mmol) and conc. HCl (3 µL, 0.03 mmol) were added to the reaction flask. The reaction was stirred at 80 °C for 72 h, and the reaction progress was monitored by TLC using an eluent condition of 10% MeOH in DCM. The reaction solvent was then removed under vacuum, and the crude product was purified by silica column chromatography (1% MeOH in DCM). This purification process yielded compound ***35*** with a yield of 34%, corresponding to 48 mg as a light brown solid. ^{1}H NMR (400 MHz, DMSO-d_6) δ 7.84 (dd, *J* = 19.8, 8.0 Hz, 2H), 7.35 (ddd, *J* = 33.5, 25.4, 7.2 Hz, 5H),

6.82 (d, *J* = 9.8 Hz, 1H), 4.45 (d, *J* = 5.9 Hz, 2H), 2.50 (s, 3H); ^{13}C NMR (100 MHz, DMSO-d_6) δ 154.11, 145.63, 142.72, 139.13, 128.80, 128.53, 127.55, 124.15, 116.74, 45.08, 9.67; ESI-MS, m/z = 240.3 $[M+H]^+$.

3-Methyl-N-phenethyl-[1,2,4]triazolo[4,3-*b*]pyridazin-6-amine (*36*)

Compound ***3*** (100 mg, 0.6 mmol) and phenylethylamine (144 mg, 1.18 mmol) were added to anhydrous EtOH (2 mL). KI (10 mg, 0.06 mmol) and conc. HCl (3 µL, 0.03 mmol) were added to the reaction flask. The reaction was stirred at 80 °C for 24 h, and the reaction progress was monitored by TLC (eluent condition: 10% MeOH in DCM). The reaction solvent was removed under vacuum, and the crude product was purified by silica column chromatography (1% MeOH in DCM). This purification process yielded compound ***36*** with a yield of 35% (52 mg). ^{1}H NMR (400 MHz, DMSO-d_6) δ 7.84 (d, *J* = 9.8 Hz, 1H), 7.45 (t, *J* = 5.4 Hz, 1H), 7.30 (d, *J* = 6.3 Hz, 4H), 7.20 (tt, *J* = 5.3, 2.2 Hz, 1H), 6.75 (d, *J* = 9.8 Hz, 1H), 3.53–3.43 (m, 2H), 2.92 (t, *J* = 7.4 Hz, 2H), 2.53 (s, 3H); ^{13}C NMR (100 MHz, DMSO-d_6) δ 154.19, 145.66, 142.68, 140.05, 129.18, 128.82, 126.60, 123.98, 116.80, 43.07, 34.44, 9.70; ESI-MS, m/z = 254.4 $[M+H]^+$.

N-(4-methoxybenzyl)-3-methyl-[1,2,4]triazolo[4,3-*b*]pyridazin-6-amine (*37*)

Compound ***3*** (100 mg, 0.6 mmol) and 4-methoxybenzylamine (162 mg, 1.2 mmol) were added to anhydrous EtOH (2 mL). KI (10 mg, 0.06 mmol) and conc. HCl (3 µL, 0.03 mmol) were added to the reaction flask. The reaction was stirred at 80 °C for 72 h, and the reaction progress was monitored by TLC (eluent condition: 10% MeOH in DCM). The reaction solvent was removed under vacuum, and the crude product was purified by silica column chromatography (1% MeOH in DCM). This purification process yielded compound ***37*** in 44% yield. ^{1}H NMR (400 MHz, DMSO-d_6) δ 7.85 (d, *J* = 9.8 Hz, 1H), 7.73 (t, *J* = 5.5 Hz, 1H), 7.37–7.32 (m, 2H), 6.93–6.89 (m, 2H), 6.80 (d, *J* = 9.8 Hz, 1H), 4.37 (d, *J* = 5.5 Hz, 2H), 3.73 (s, 3H), 2.52 (s, 3H); ^{13}C NMR (100 MHz, DMSO-d_6) δ 158.91, 154.04, 145.65, 142.71, 130.95, 129.93, 124.03, 116.80, 114.18, 55.52, 55.36, 44.54, 9.69; ESI-MS, m/z = 270.4 $[M+H]^+$.

3-((3-Methyl-[1,2,4]triazolo[4,3-*b*]pyridazin-6-yl)amino)-N-phenylpropanamide (*38*)

Compound ***28*** (200 mg, 0.9 mmol), TEA (367 mg, 3.61 mmol) and HATU (516 mg, 1.37 mmol) were dissolved sequentially in acetonitrile (3 mL) and stirred for approximately 5 min. Aniline (91 µL, 1.0 mmol) was added dropwise into the reaction mixture and stirred for 3 h at RT, and the reaction progress was monitored by TLC (eluent condition: 15% MeOH in DCM). The reaction was quenched by adding H_2O (5 mL) and partitioned with ethyl acetate (15 mL), and the organic layer was washed with brine (10 mL). The organic layer was collected, dried over anhydrous Na_2SO_4, filtered, concentrated, and purified by silica column chromatography (6% MeOH in DCM). This purification process yielded

compound ***38*** with a yield of 17% (46 mg). ^{1}H NMR (400 MHz, DMSO-d_6) δ 9.95 (s, 1H), 7.84 (d, J = 9.8 Hz, 1H), 7.60 (d, J = 8.0 Hz, 2H), 7.47 (t, J = 5.6 Hz, 1H), 7.29 (t, J = 7.7 Hz, 2H), 7.03 (t, J = 7.5 Hz, 1H), 6.78 (d, J = 9.9 Hz, 1H), 3.58 (q, J = 6.3 Hz, 2H), 2.71 (t, J = 6.6 Hz, 2H); ^{13}C NMR (100 MHz, DMSO-d_6) δ 170.04, 154.21, 145.64, 142.70, 139.67, 129.13, 123.97, 123.54, 119.52, 116.88, 37.53, 35.60, 14.66, 9.71; ESI-MS, m/z= 297.5 $[M+H]^+$.

N-(2-((3-methyl-[1,2,4]triazolo[4,3-*b*]pyridazin-6-yl)amino)ethyl)benzamide (*39*)

Compound ***32*****a** (150 mg, 0.65 mmol) and anhydrous pyridine (2 mL) were dissolved together and cooled to 0 °C. Benzoyl chloride (90 mg, 0.64 mmol, 75 μL) was added dropwise to the reaction flask, and the reaction was performed with stirring for 4 h at RT. The reaction progress was monitored by TLC (eluent condition: 10% MeOH in DCM). Subsequently, the reaction solvent was removed under vacuum, and the crude product was purified by silica column chromatography (3% MeOH in DCM). This purification process yielded compound ***39*** with a yield of 33% (63 mg). ^{1}H NMR (400 MHz, DMSO-d_6) δ 8.79 (s, 1H), 8.29 (d, J = 44.8 Hz, 1H), 8.07–7.75 (m, 3H), 7.47 (d, J = 26.7 Hz, 3H), 7.17–6.96 (m, 1H), 3.57 (s, 4H), 2.59 (s, 3H). ^{13}C NMR (100 MHz, DMSO-d_6) δ 166.80, 155.23, 134.77, 131.66, 128.69, 127.73, 122.65, 120.46, 79.79, 41.09, 38.25, 9.40; ESI-MS, m/z = 297.5 $[M+H]^+$.

1-(2-((3-Methyl-[1,2,4]triazolo[4,3-*b*]pyridazin-6-yl)amino)ethyl)-3-phenylurea (*40*)

Compound ***32*****a** (100 mg, 0.43 mmol) and DIPEA (113 mg, 0.87 mmol) were mixed in acetonitrile (1 mL) and stirred for 5 minutes at RT. Phenylisocyanate (57 mg, 0.48 mmol, 53 μL) was added dropwise into the reaction flask and then stirred at RT for 5 h. The reaction progress was monitored by TLC (eluent condition: 15% (10% ammonia in MeOH) in DCM). The reaction mixture was filtered, and the product was washed with cold acetonitrile. This purification process yielded compound ***40*** with a yield of 44%, corresponding to 60 mg as a white solid. ^{1}H NMR (400 MHz, DMSO-d_6) δ 8.52 (s, 1H), 7.86 (d, J = 9.8 Hz, 1H), 7.48–7.34 (m, 3H), 7.21 (t, J = 7.8 Hz, 2H), 6.92–6.85 (m, 1H), 6.76 (d, J = 9.8 Hz, 1H), 6.26 (d, J = 5.4 Hz, 1H), 3.42–3.36 (m, 4H), 2.51 (s, 3H). ^{13}C NMR (100 MHz, DMSO-d_6) δ 155.84, 154.43, 145.67, 142.71, 140.92, 129.09, 124.03, 121.52, 118.15, 116.80, 41.67, 38.42, 9.66; ESI-MS, m/z = 312.5 $[M+H]^+$.
